# Tailoring nanoscale interfaces for perovskite–perovskite–silicon triple-junction solar cells

**DOI:** 10.1038/s41565-025-02015-x

**Published:** 2025-10-07

**Authors:** Jianghui Zheng, Guoliang Wang, Leiping Duan, Weiyuan Duan, Yang Jiang, Phoebe Pearce, Yijun Gao, Md Arafat Mahmud, Chwenhaw Liao, Tik Lun Leung, Jueming Bing, Zhuofeng Li, Zhenyu Sun, Xin Cui, Christopher Bailey, Marko Jankovec, Jianpeng Yi, Runmin Tao, Lijie Zheng, Baihong Zhu, Yue Sun, Nan Sun, Gaosheng Huang, Li Wang, Andreas Lambertz, Stephen Bremner, Xinqin Liao, Tingzhu Wu, Guohua Xie, Mathias Uller Rothmann, Marko Topič, David R. McKenzie, Kaining Ding, Wei Li, Zhong Chen, Anita W. Y. Ho-Baillie

**Affiliations:** 1 School of Physics, The University of Sydney, Sydney, New South Wales Australia; 2 Sydney Nano, The University of Sydney, Sydney, New South Wales Australia; 3 Australian Centre for Advanced Photovoltaics (ACAP), University of New South Wales, Sydney, New South Wales Australia; 4 School of Electronic Science and Engineering, Xiamen University, Xiamen, China; 5 Shenzhen Hiking PV Technology Limited, Shenzhen, China; 6 IMD-3 Photovoltaics, Forschungszentrum Jülich GmbH, Jülich, Germany; 7 State Key Laboratory of Advanced Technology for Materials Synthesis and Processing, Wuhan University of Technology, Wuhan, China; 8 Foshan Xianhu Laboratory of the Advanced Energy Science and Technology Guangdong Laboratory, Xianhu Hydrogen Valley, Foshan, China; 9 School of Engineering, The Australian National University, Canberra, Australian Capital Territory Australia; 10 Faculty of Electrical Engineering, University of Ljubljana, Ljubljana, Slovenia; 11 Institute of Flexible Electronics (IFE, Future Technologies), Institute of Future Display Technology, Tan Kah Kee Innovation Laboratory, Xiamen University, Xiamen, China; 12 Present Address: Yuhuan Jinko Solar Co., Ltd, Yuhuan, China

**Keywords:** Devices for energy harvesting, Electronic devices, Electronic properties and materials

## Abstract

Triple‐junction solar cells theoretically outperform their double-junction and single‐junction counterparts in power conversion efficiency, yet practical perovskite–perovskite–silicon devices have fallen short of both theoretical limits and commercial targets. To address surface defects in the top perovskite junction, we introduce a piperazine-1,4-diium chloride treatment, which replaces less stable lithium fluoride. For interfacing the top and middle perovskite junctions, we optimize the size of gold nanoparticles deposited on atomic layer-deposited tin oxide for best ohmic contacting with minimal optical losses. Applying these strategies, our champion 1-cm^2^ triple‐junction cell achieved a third party-verified reverse‐scan power conversion efficiency of 27.06% with an open circuit voltage of 3.16 V. Scaling up to 16 cm^2^, the device produced a certified steady‐state power conversion efficiency of 23.3%. Device longevity also improved by eliminating methylammonium and incorporating rubidium into the perovskite bulk alongside the piperazine-1,4-diium chloride surface layer. An encapsulated 1-cm^2^ cell retained 95% of its initial efficiency after 407 h at maximum power point and passed the IEC 61215 thermal cycling test. These results represent advancements towards efficient and stable perovskite–perovskite–silicon triple-junction solar cells.

## Abstract (web summary)

‘Ultrathin’ gold interlayers are shown to form nanoparticles that balance optical losses and electrical contact, enabling efficient perovskite–perovskite integration for scalable and durable triple-junction photovoltaics.

## Main

To accelerate the transition towards a net-zero-CO_2_-emission economy, improving the efficiency of solar cells is instrumental in reducing the levelized cost of energy of photovoltaic systems, provided device lifetime can be maintained. Multi-junction solar cells that stack semiconductor materials with descending bandgap from top (sun-facing) to bottom convert sections of the solar spectrum more efficiently. They therefore have a high theoretical power conversion efficiency limit: ~45% for double-junction and ~51% for triple-junction cells^1^ when there is no constraint on the choice of materials and, therefore, of their bandgaps. Metal halide perovskite solar cells are potentially cost-effective^1^, and their rapid efficiency improvements and ease of fabrication make them highly attractive to be partnered with photovoltaic materials, such as perovskites or silicon (Si), for multi-junction tandem devices. The ease of fabrication is conducive to monolithic integration, whereby one junction is directly fabricated onto another, or the junctions are electrically bonded^2^. Monolithic integration is particularly useful for triple-junction tandem devices resulting in two-terminal connections as opposed to four-terminal or six-terminal connections^1^.

Great progress has been made in both all-perovskite and perovskite-on-Si triple-junction devices. The first all-perovskite (perovskite–perovskite–perovskite) triple-junction cell was reported in 2019, with a power conversion efficiency (PCE) of 6.7% (ref. ^3^). In just 5 years, the efficiency significantly improved, to 28.7% most recently^4^. In terms of perovskite on the Si (perovskite–perovskite–Si) triple junction, the first demonstration was reported by Werner et al.^5^ at 14.0% efficiency in 2018, by integrating perovskite–perovskite double junctions onto a front- and rear-side textured Si bottom cell. Both perovskite layers were fabricated via a two-step method combining thermal evaporation and spin-coating^5^. After a hiatus of four years, a second report was the first to reach the 20% efficiency milestone using a solution-processed self-assembled monolayer as the hole transport layer for the perovskite cells^6^. Since then, higher PCEs have been reported, such as the most recent one certified at 27.1% (Supplementary Table 1). Over time, the stability of these cells has also improved (Supplementary Table 1), from minutes for the earlier unencapsulated devices to hundreds of hours for encapsulated devices under maximum power point tracking (MPPT). Despite the progress made, there is ample scope for improving the stability and performance of perovskite-on-Si triple junctions to fully realize their potential.

Many of the demonstrated perovskite–Si triple-junction devices (Supplementary Table 1) contain methylammonium (MA) and lithium fluoride (LiF), which are known to cause cell instability^7,8^. To replace LiF between the perovskite and the C_60_ layer, a piperazine-1,4-diium (also known as piperazinium)-based material can be considered. Piperazinium di-iodide has been shown to effectively passivate surface defects in 1.26-eV (ref. ^9^) and 1.55-eV (ref. ^10^) or 1.56-eV (ref. ^11^) perovskites. Additionally, it regulates band bending, facilitating efficient charge extraction.

Regarding monolithic integration of the top two perovskite junctions, ‘ultrathin’ gold (Au) combined with atomic layer-deposited (ALD) SnO_2_ has been commonly used^12–17^. At a certain thinness, for example, ~1 nm (refs. ^12–15,18,19^) and ~0.4 nm (ref. ^16^), the ‘ultrathin’ Au takes the form of nanoparticles. While this concept has been discussed by Tian et al.^20^ in the context of n–i–p perovskite–organic double-junction tandem cells, this has not been considered before in the context of p–i–n cells.

In this paper, we investigate the effect of rubidium (Rb) incorporation in improving the performance and stability of MA-free wide-bandgap (1.91 eV) perovskite and developed piperazinium di-chloride (PDCl) surface treatment, replacing LiF from the 1.91-eV high bromide-containing perovskite film. For integrating the top two perovskite junctions, we investigate the relationship between nanoparticle size and coverage with deposition time, including the critical ‘thickness’ at which the particles begin to form clusters or a semicontinuous film, and the pathway for minimizing optical loss without compromising electrical performance. Using these strategies, we demonstrate a 1-cm^2^ champion perovskite–perovskite–silicon triple-junction device producing a third party-verified reverse-scan PCE of 27.06%, with an open circuit voltage (*V*_OC_) of 3.16 V. A large-area, 16 cm^2^, champion perovskite triple-junction solar cell produced a certified steady-state PCE of 23.3%. In terms of stability, a 1-cm^2^ encapsulated cell maintained 95% of its initial efficiency after 407 h MPPT under continuous light illumination. Additionally, an encapsulated perovskite triple-junction solar cell passed the International Electrotechnical Commission (IEC) 61215 thermal cycling test after 200 cycles between −40 °C and 85 °C.

## Surface treatment for the perovskite top junction

**Fig. 1:**
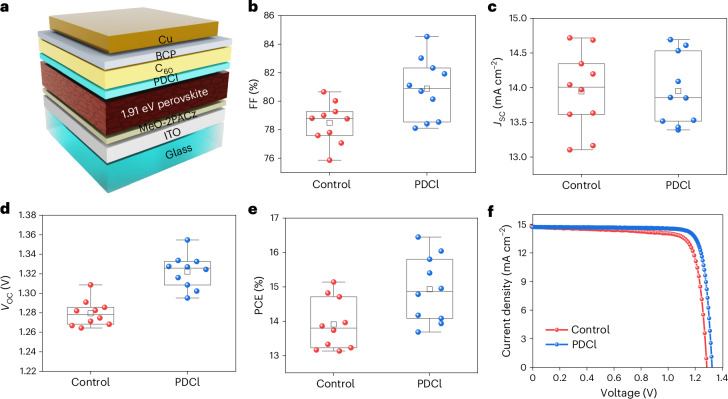
Photovoltaic performance of wide-bandgap perovskite solar cell. **a**–**e**, Schematic (**a**) and distributions of FF (**b**), *J*_SC_ (**c**), *V*_OC_ (**d**) and PCE (**e**) of control and PDCl-treated 1.91-eV wide-bandgap perovskite p–i–n devices (10 devices in each type). Top value: maximum; top bar: 75th percentile; middle bar: median; open squares: mean; bottom bar: 25th percentile; lowest value: minimum; solid circles: measured data. **f**, Reverse-scan current density–voltage (*J–V*) curves of the champion device in each group.

The schematic of the wide-bandgap (1.91 eV) perovskite junction is shown in Fig. 1a. The cell structure consists of glass/ITO/MeO-2PACz/Cs_0.16_Rb_0.04_FA_0.8_Pb(I_0.45_Br_0.55_)_3_/(PDCl)/C_60_/BCP/Cu, where FA (formamidinium) and MeO-2PACz ([2-(3,6-dimethoxy-9*H*-carbazol-9-yl)ethyl] phosphonic acid) are used as a hole transport layer. Details of cell fabrication can be found in Methods. The results of Rb incorporation optimization can be found in Supplementary Figs. 1 and 2. It was found that 4% Rb added into the perovskite precursor results in optimum device performance (Supplementary Fig. 1a–d). This correlates with trends observed in the X-ray diffraction patterns of the corresponding films, whereby 4% Rb incorporation results in the minimum PbI_2_ and non-perovskite-phase (labelled ‘δ’ in Supplementary Fig. 1f) peaks when compared to films with lower or higher amounts of Rb (Supplementary Fig. 1f). The presence of Rb in the perovskite layer is also evident by time-of-fight secondary ion mass spectrometry (Supplementary Fig. 1g).

To further improve the performance of the wide-bandgap Cs_0.16_Rb_0.04_FA_0.8_Pb(I_0.45_Br_0.55_)_3_ perovskite, a PDCl surface treatment was applied (Fig. 1a). Supplementary Fig. 3 shows the molecular structure and electrostatic potential of PD^+^ as well as a top-view scanning electron microscopy (SEM) image of PDCl-treated perovskite. Figure 1b–e and Supplementary Table 2 show improvements in average *V*_OC_, from 1,280 mV to 1,322 mV, average fill factor (FF), from 0.78 to 0.81, and average PCE, from 13.7% to 15.1%, after PDCl treatment. The short circuit current density (*J*_SC_) did not improve (Fig. 1c) due to insignificant change in the perovskite layer absorbance (Supplementary Fig. 4) and bandgap. The latter remains at 1.91 eV after PDCl treatment (Supplementary Fig. 5b). Nevertheless, respectable performance was achieved by the champion PDCl-treated device achieving a record FF of 84.5%, while the best FF for a 1.91 eV perovskite reported so far is 83.5% (ref. ^17^). For comparison, devices that replace bathocuproine (BCP) with ALD SnO_2_ are also demonstrated, as this cell structure (Supplementary Fig. 6a, right) was used in later tandem demonstrations in this work. The results show a higher average and narrower distribution in all cell parameters, including power conversion efficiencies (Supplementary Fig. 6b–e) in SnO_2_-based devices compared to BCP-based ones.

**Fig. 2:**
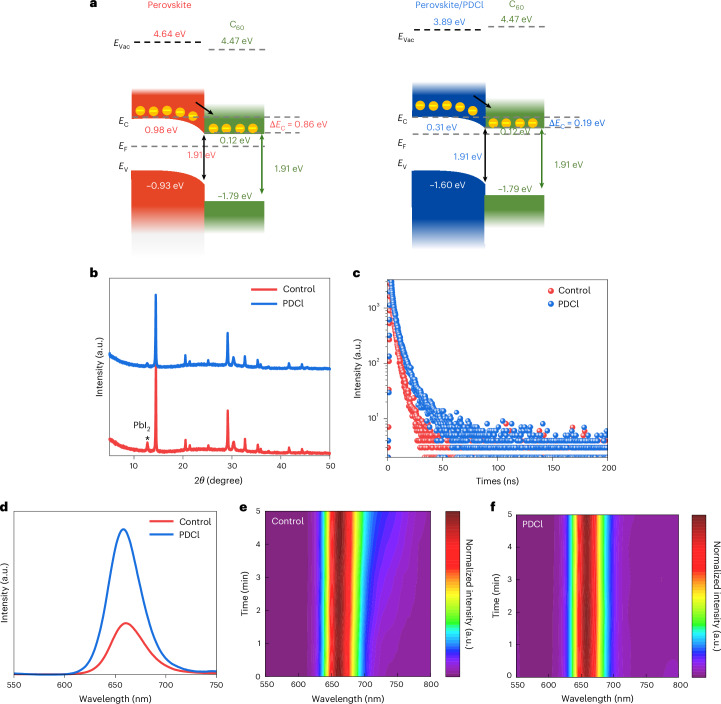
Film characterization of wide-bandgap perovskite. **a**, Energy band diagrams of the perovskite/PDCl interface. *E*_Vac_, energy levels for the vacuum level; *E*_C_, conduction band edge; *E*_V_, valence band edge; *E*_F_, Fermi level. **b**, X-ray diffraction measurements of untreated (red) and PDCl-treated (blue) perovskites. **c**–**f**, TRPL (**c**), steady-state PL (**d**) and their time evolutions of representative untreated (red) (**e**) and PDCl (blue)-treated (**f**) perovskites. a.u., arbitrary units.

The reasons for the performance improvement are many. Results from ultraviolet photoelectron spectroscopy (UPS) (Supplementary Fig. 7) revealed a reduction in band offsets between the perovskite and the C_60_ layer after PDCl surface treatment for both the valence and conduction bands (Fig. 2a), enhancing both the hole and electron transport. Enhanced carrier transport can be seen in the results for transient photocurrent measurements (Supplementary Fig. 8), which led to lowered series resistance (*R*_S_) (Supplementary Fig. 9a) and increased FF (Fig. 1b) in PDCl-treated cells.

X-ray diffraction revealed the reduction of PbI_2_ peak intensity (Fig. 2b) after PDCl treatment. Although the (100) peak intensity decreased slightly (Supplementary Fig. 10a), the PbI_2_/(100) intensity ratio (Supplementary Fig. 10b) also decreased, probably as a result of the reaction between PDCl and PbI_2_ (ref. ^11^) present on the surface of untreated perovskite film (Supplementary Figs. 1f and 2e). Results from X-ray photoelectron spectroscopy (XPS) also revealed reductions in Pb0 peaks at 141.8 eV and 137.1 eV in the PDCl-treated perovskite (Supplementary Fig. 11) compared to the control (untreated) film.

As a result, the trap densities in PDCl-treated perovskite cells are reduced, as seen in Supplementary Fig. 12a, for energy levels ~0.35 eV away from the valence band (*E*_V_), as determined from thermal admittance spectroscopy and Mott–Schottky analysis. The built-in potential (*V*_bi_) determined for PDCl-treated cells is also higher than that of untreated cells (Supplementary Fig. 12b). This aligns with the trend observed for increased *V*_OC_ (Fig. 1d) and increased steady-state photoluminescence (PL) intensity (Fig. 2c and Supplementary Fig. 13) in PDCl-treated cells and films, respectively. Such defect passivation improved the photogenerated carrier lifetime (Supplementary Table 3) determined from time-resolved photoluminescence (TRPL) (Fig. 2d). Finally, results of temperature-dependent *V*_OC_ measurements (Supplementary Fig. 14) show that the activation energy for the recombination current (*E*_a_) dropped from 1.90 eV for the untreated device to 1.79 eV for the PDCl-treated device, in which the recombination mechanism became more bulk-dominated, which in turn suggests an improved perovskite/C_60_ interface. Therefore, these combined factors contribute to an improved voltage output and FF for PDCl-treated cells, as shown in Fig. 1.

Furthermore, the time evolution of steady-state PL in Supplementary Fig. 15 shows suppressed phase segregation in Rb-incorporated wide-bandgap perovskites, and even more so after additional PDCl treatment (compare Fig. 2e,f), indicating the effectiveness of the strategies developed in this work for improving photostability for wide-bandgap perovskites.

## Interface modulation for integrating perovskite junctions

**Fig. 3:**
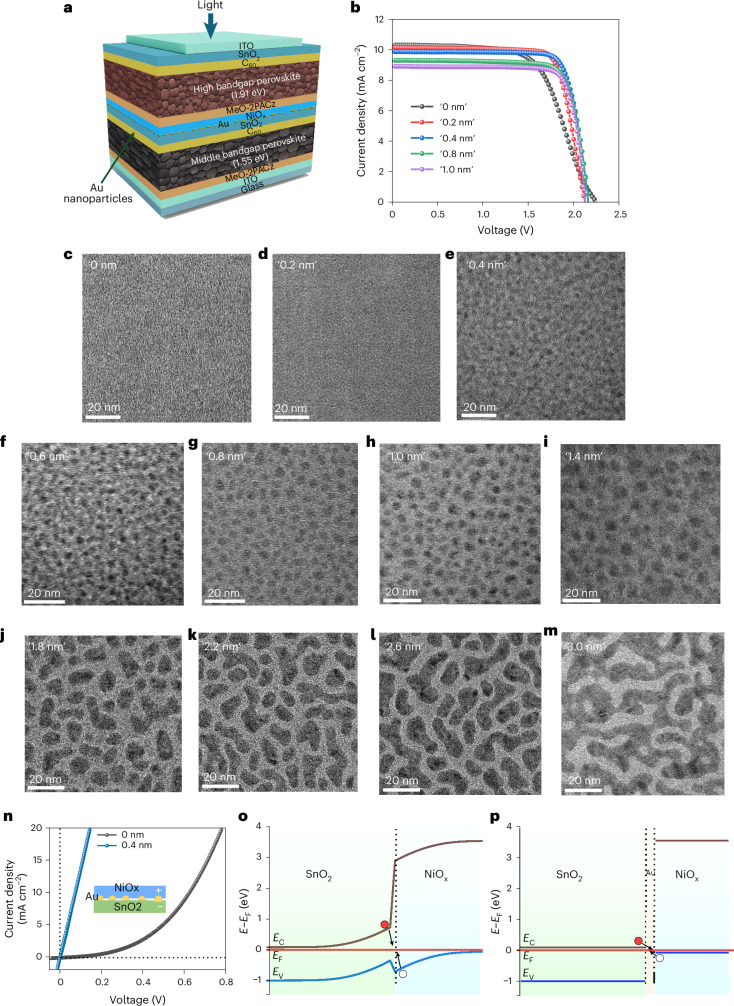
Au nanoparticle interface optimization for the perovskite–perovskite double junction. **a**,**b**, Device structure schematic (**a**) and *J*–*V* curves (**b**) of rudimentary 1.55 eV perovskite–1.91 eV perovskite double-junction semitransparent test solar cells, without Rb incorporation or PDCl treatment, for evaluating the effect of different Au deposition times. **c**–**m**, TEM micrographs of Au deposited for different durations (using a nominal thickness reading from the thermal evaporator): ‘0 nm’ (**c**); ‘0.2 nm’ (**d**); ‘0.4 nm’ (**e**); ‘0.6 nm’ (**f**); ‘0.8 nm’ (**g**); ‘1.0 nm’ (**h**); ‘1.4 nm’ (**i**); ‘1.8 nm’ (**j**); ‘2.2 nm’ (**k**); ‘2.6 nm’ (**l**); ‘3.0 nm’ (**m**). **n**, Dark *J*–*V* characteristics of the SnO_2_/(Au)/NiO_*x*_ testing structure. **o**,**p**, Simulated band diagram of the SnO_2_/(Au)/NiO_*x*_ stack at equilibrium without (**o**) and with (**p**) Au at the interface.

To better understand the properties of ‘ultrathin’ Au on the ALD SnO_2_ layer, to further improve the perovskite–perovskite interface for monolithic perovskite tandems, we systematically carried out transmission electron microscopy (TEM) of test structures, as shown in Supplementary Fig. 16. The measured TEM diffraction rings corresponding to Au and SnO_2_ on amorphous carbon are shown in Supplementary Fig. 17. It was found that ‘0.4 nm’ Au, as read out by the thickness monitor of the thermal evaporator, is, in fact, not a continuous film but takes the form of distributed nanoparticles (Fig. 3e and Supplementary Fig. 16). As deposition times increased, so did the nanoparticle coverage (Fig. 3d–i). Supplementary Table 5 lists the nanoparticle average size, coverage and spacing with deposition time. Clusters began to form when the nominal ‘thickness’ was ≥‘1.8 nm’ (Fig. 3j–l). At ‘3.0 nm’, the deposited Au became a semicontinuous film (Fig. 3m).

We then carried out rigorous coupled-wave analysis (RCWA) simulations (see Methods for details). Unit cells (Supplementary Fig. 18) with Au coverage from Supplementary Table 5 for nominal thicknesses of ‘0.2’, ‘0.4’, ‘0.6’, ‘0.8’, ‘1.0’ and ‘3.0 nm’ were generated, beginning with a simple Au/SnO_2_/ITO/glass test structure (Supplementary Fig. 19) for model verification. Simulated transmittance of Au nanoparticles with various coverages or an Au planar film on SnO_2_(20 nm)/ITO/glass are shown in Supplementary Fig. 19a,b, while the measured results are shown in Supplementary Fig. 19c. Comparing with the results for a planar layer of Au (Supplementary Fig. 19b), a dip in transmittance around 600 nm is seen in the experimental measurements (Supplementary Fig. 19c), which was replicated by optical simulations only in the case of Au nanoparticles (Supplementary Fig. 19a). This indicates that the optical absorption in the Au nanoparticles was caused in part by localized surface plasmon resonances in the nanoparticles^21^. We then optically modelled a semitransparent perovskite–perovskite tandem as a function of the amount of Au deposited. Rudimentary experimental perovskite–perovskite tandems (Fig. 3a and Supplementary Table 4) were fabricated without Rb incorporation and PDCl treatment to illustrate the effect of varying the amount of Au. Results of optical modelling of a semitransparent perovskite–perovskite tandem as a function of Au (Supplementary Fig. 20) show that Au nanoparticles at the interface were primarily responsible for parasitic absorption, with ‘thicker’ Au or more of the Au nanoparticles causing an increase in parasitic absorption. This prediction explains the drop in the observed current output of the demonstrated tandem devices (Fig. 3b and Supplementary Table 4).

The simulated absorptance (Supplementary Fig. 21a) and *J*_SC_ (Supplementary Fig. 21b) indicate that the minimum amount of interfacial Au produces the highest current output. However, the presence of Au is essential to facilitate ohmic contact as its absence results in an ‘S’ shape for the *J–V* curve (Fig. 3b) or a nonlinear response in the dark *J*–*V* curve (Fig. 3n). Results of simulations (Fig. 3o,p) show that the presence of Au at the SnO_2_/NiO_*x*_ interface, even at the local level (for example, in the form of nanoparticles), suppressed band bending, thereby lowering the barrier of the carrier recombination junction between the subcells.

## Triple-junction demonstration

**Fig. 4:**
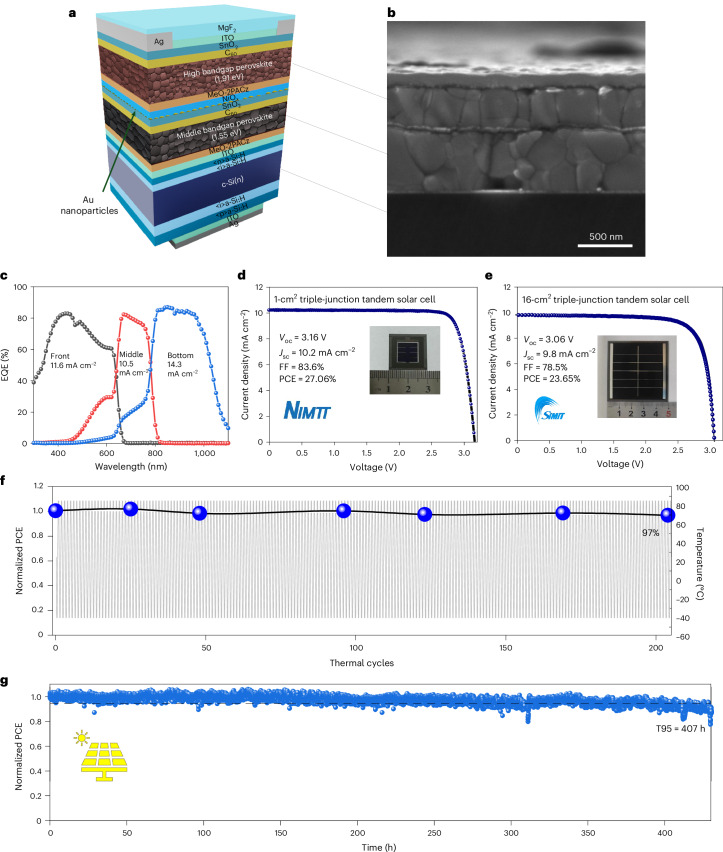
Photovoltaic performance and stability of the triple-junction perovskite–perovskite–silicon tandem. **a**–**c**, Device structure schematic (**a**), cross-sectional SEM image (**b**) and EQE (**c**) curves (black for wide-bandgap perovskite; red for mid-bandgap perovskite; and blue for low-bandgap silicon) of a representative triple-junction tandem. **d**,**e**, Third party-measured reverse-scan *J*–*V* curves of champion triple junctions: 1 cm^2^ (**d**) and 16 cm^2^ (**e**). **f**,**g**, Encapsulated 1-cm^2^ triple-junction solar cells under thermal cycling (between −40 °C and ~85 °C) (**f**) and MPPT (**g**) at 25 °C in ambient. T95, time for the power output to decrease to 95% of its initial value.

For monolithic perovskite–perovskite–Si triple-junction (Fig. 4a) tandem demonstrations, we applied Rb incorporation and PDCl treatment strategies to the top perovskite junction and the Au nanoparticle interfacing strategy to the top and middle junctions. The SEM cross-section of a triple-junction cell is shown in Fig. 4b and details of its fabrication can be found in Methods.

For reference, the *J*–*V* curve of an optimized 1.55-eV perovskite–1.91-eV perovskite double-junction semitransparent test cell is shown in Supplementary Fig. 22 producing a higher *V*_OC_ than those in Fig. 3, thus showing the effectiveness of the strategies. For comparison, triple junctions that replace Au nanoparticles with 15-nm ITO were also fabricated. Results show that while current outputs are comparable (Supplementary Fig. 23b), Au nanoparticle interfacing is more effective than 15-nm ITO in producing higher and narrower distributions of FF (Supplementary Fig. 23a) and *V*_OC_ (Supplementary Fig. 23b), resulting in higher performance (Supplementary Fig. 23d). This is due to the better ability of Au nanoparticles to localize any shunting effects while still facilitating local ohmic contact for vertical carrier transport, compared with 15 nm ITO, which is more conductive laterally^22^.

The champion 1-cm^2^ triple-junction device achieved a third party-verified reverse-scan PCE of 27.06% (Fig. 4d and Supplementary Fig. 24). For reference, in-house measurement results are shown in Supplementary Fig. 25. The efficiency and *V*_OC_ (3.16 V) achieved are among the highest to date for triple-junction perovskite-on-silicon tandem solar cells (Supplementary Fig. 26 and Supplementary Table 1). The external quantum efficiency (EQE) and the integrated *J*_SC_ values from the three subcells (Fig. 4c) show that the top and middle perovskite junctions are still current-limiting, requiring further improvement in future work, including red-shifting the middle cell absorption edge by introducing tin in the absorber. Future work will use a front textured silicon bottom cell to enhance light-trapping and to reduce reflections from the top and middle perovskite junctions.

To demonstrate the suitability of interfacing designs developed in this work for large areas, a 16-cm^2^ tandem device was demonstrated (Fig. 4e), which is at least an order of magnitude larger than perovskite triple junctions reported so far^3,20^ (Supplementary Table 1). The champion device achieved a certified reverse-scan PCE of 23.65% and a steady-state (300 s) PCE of 23.3% (Supplementary Fig. 27). A respectable FF of 0.78, for such a large-area device, was achieved, partly due to the ability of the Au nanoparticles to localize large-area film, non-uniformity related shunts while still facilitating vertical carrier transport. While the drop of *V*_OC_ on the large area was not significant, future improvements will require optimization of the front grid design to minimize shading and to improve *J*_SC_ and the use of area-scalable perovskite deposition methods to improve the FF to ≥80%.

To evaluate the stability of the 1-cm^2^ triple-junction tandems, devices were encapsulated before IEC 61215 thermal cycling and MPPT tests. Due to optical loss from encapsulation, the PCEs of the devices were 25.1% and 25.2%, respectively, before thermal cycling and MPPT. One device maintained 97% of its initial PCE after 200 thermal cycles (Fig. 4f), passing the IEC 61215 thermal cycling test. The second device maintained 95% of its PCE after 407 h of continuous 1-Sun illumination (Fig. 4g). These stabilities exceed those of reported perovskite triple junctions^23^, including perovskite–perovskite–silicon triple junctions (Supplementary Table 1). Such improved stability is due to the presence of Rb (suppressing phase segregation as shown in Supplementary Fig. 15 and discussed previously) and the absence of MA in our triple junctions. To illustrate this, light and thermal stability tests were performed on Cs_0.2_FA_0.8_Pb(I_0.45_Br_0.55_)_3_ perovskite cells, Cs_0.16_Rb_0.04_FA_0.8_Pb(I_0.45_Br_0.55_)_3_ perovskite cells and Cs_0.16_MA_0.04_FA_0.8_Pb(I_0.45_Br_0.55_)_3_ perovskite cells (Supplementary Fig. 28). It was found that Rb-incorporated and MA-free wide-bandgap devices (Supplementary Fig. 28b,f) degrade less, or are more stable, than Rb-free and MA-free devices (Supplementary Fig. 28a,e), which in turn are more stable than Rb-free and MA-containing devices (Supplementary Fig. 28c,g). While the latter two types of perovskite have been used as top cells in high-performance triple-junction tandem cells^24–26^, when both performance and stability are considered, Rb-incorporated, MA-free perovskites are highly suitable. Another reason for the respectable stability is the replacement of LiF by PDCl at the perovskite/C_60_ interface. Although LiF has been used in many state-of-the-art triple junctions (Supplementary Table 1), LiF is known to reduce device stability^27^. PDCl also provides additional phase-segregation suppression, as shown in Fig. 2f and discussed previously. Results in Supplementary Fig. 28 also show that PDCl treatment for Rb-incorporated, MA-free perovskites is desirable for both light (Supplementary Fig. 28d) and thermal (Supplementary Fig. 28h) stability.

## Conclusions

In summary, we report improved interface designs for the perovskite–perovskite–Si triple junction via the use of piperazine-1,4-diium chloride surface treatment for the 1.91-eV Rb-incorporated, MA-free and LiF-free top perovskite cell, and the use of Au nanoparticles for monolithic perovskite–perovskite integration, producing improved performance and stability. The form of the ‘ultrathin’ Au is clarified, and consists of nanoparticles. The relationship between nanoparticle size and coverage has been systemically investigated for a better design of the perovskite–perovskite interface to improve device performance. A third party-verified reverse-scan PCE of 27.06% was achieved for the champion perovskite–perovskite–silicon triple junction at 1 cm^2^. A certified steady-state PCE of 23.3% was achieved for 16 cm^2^. An encapsulated triple junction maintained 95% of its initial efficiency after 407 h of MPPT and another encapsulated triple junction passed the IEC 61215 thermal cycling test. These results serve as the foundation for developing more efficient and stable perovskite–perovskite–silicon triple-junction solar cells in the future, with improved electrical current from the top and middle perovskite junctions via bandgap engineering and light-trapping. Improvements in large-area film depositions and front grid design are also important for future large-area triple junctions, to produce voltage and current outputs and fill factors comparable to state-of-the-art small-area devices.

## Methods

### Materials

Formamidinium iodide (FAI), formamidinium bromide (FABr), methylammonium iodide (MAI) and PDCl were sourced from GreatCell Solar Materials. Lead iodide (PbI_2_), lead bromide (PbBr_2_), rubidium iodide (RbI) and MeO-2PACz were purchased from Tokyo Chemical Industry. BCP was purchased from Lumtec. C_60_ was purchased from Nano C. Tetrakis(dimethylamino)tin(IV) (TDMASn) was purchased from Strem Chemicals. Gold, copper and silver shot were purchased from ESPI Metals. Other dry chemicals were purchased from Sigma-Aldrich and all solvents were purchased from Alfa Aesar. Pre-patterned indium tin oxide (ITO) glasses with a sheet resistance of 8 Ω sq^−1^ were purchased from Wuhan Jinge Solar Energy Technology.

### Device fabrication

For the fabrication of the silicon heterojunction bottom solar cell, 6-inch N-type polished Czochralski wafers with a thickness of 150 μm and a resistivity ranging from 1 to 5 Ω cm were used. A wet-chemical process, including saw-damage removal and cleaning, was applied to the as-cut wafers. No texturing process was used for this wafer. Subsequently, an intrinsic a-Si:H passivation layer (~5 nm) was first deposited by plasma-enhanced chemical vapour deposition (PECVD) on both sides of the wafer. Then, n-type (~5 nm) and p-type (~8 nm) a-Si:H layers were sequentially deposited at the front and back sides of the wafer, respectively. After PECVD, the back contact of the silicon cells was fabricated by stacking sputtered ITO (80 nm) and then thermally evaporated Ag through a shadow mask with an opening of 1.1 × 1.1 cm^2^ or 4.1 × 4.1 cm^2^ on the rear side. For the front side, a 20-nm-thick ITO layer was deposited on the n front side through a shadow mask of 1.1 × 1.1 cm^2^ or 4.1 × 4.1 cm^2^, defining the aperture area of the silicon bottom cell and acting as a recombination layer between the silicon bottom cell and the perovskite middle cell. The silicon bottom cells were then laser-cut to a 2 × 2 cm^2^ or 5 × 5 cm^2^ square substrate for small-area (1 cm^2^) and large-area (16 cm^2^) tandem fabrication.

The 1.55-eV middle perovskite has the structure: MeO-2PACz/Cs_0.08_Rb_0.02_FA_0.9_PbI_3_/ C_60_/SnO_2_/Au.

The front of the silicon solar cell was first treated with ultraviolet–ozone (UVO) cleaner for 5 min. This was followed by deposition of the hole transport layer MeO-2PACz (0.5 mg ml^−1^ in menthol) via spin-coating at 4,000 rpm for 20 s, followed by annealing at 95 °C for 10 mins. The 1.5 M Cs_0.08_Rb_0.02_FA_0.9_PbI_3_ precursor was then spin-coated at 2,000 rpm for 20 s, followed by 6,000 rpm for 30 s. N_2_ was blown onto the surface in the last 20 s before the end of the spin process. The film was annealed at 105 ^o^C for 10 min, producing a deep, dark, dense perovskite film. The substrates were then transferred into a thermal evaporation chamber for 20 nm C_60_ deposition. This was followed by 20-nm SnO_2_ deposition by thermal ALD in an Arradiance GEMStar reactor. TDMASn was used as the Sn precursor and was held at 60 °C in a stainless-steel container. Water was used as an oxidant and was delivered from a stainless-steel container at room temperature, and the precursor delivery manifold temperature was set to 115 °C. The TDMASn/purge1/H_2_O/purge2 times were 1 s/10 s/0.2 s/15 s with corresponding nitrogen flows of 30 sccm/90 sccm/90 sccm/90 sccm to the deposition chamber at 80 °C. A 20-nm tin oxide layer was formed after 135 cycles. After that, Au was deposited via thermal evaporation for different durations. Nominal thickness reading at 0, 0.2, 0.4, 0.6, 0.8 and 1 nm (using the Inficon quartz crystal monitor with corrected tooling factor for gold material) was used to distinguish different deposition times.

The 1.91-eV top perovskite cell had the structure: NiO_*x*_/MeO-2PACz/Cs_0.16_Rb_0.04_FA_0.8_Pb(I_0.45_Br_0.55_)_3_/(PDCl)/C_60_/SnO_2_/Ag/MgF_2_. The 10-nm NiO_*x*_ was deposited by sputter-coating using a 2-inch target under 60-W radiofrequency power in Ar at 2 mTorr using an AJA International sputtering system. The same concentration of MeO-2PACz was used as above for deposition, except for a longer duration of 30 s followed by annealing at a higher temperature of 100 °C. A 0.8 M, wide-bandgap perovskite precursor was then spin-coated using a single-step spin programme (3,000 rpm for 50 s), with nitrogen gas blown onto the surface during the last 25 s of the spin process. The resulting film was annealed at 105 °C for 10 min, yielding a deep red, dense film. The value of *x* in Cs_0.2−*x*_Rb_*x*_FA_0.8_Pb(I_0.45_Br_0.55_)_3_ was allowed to vary between 0 and 0.12 for optimizing perovskite film quality and device performance. The results can be found in Supplementary Figs. 1 and 2. For the PDCl-treated perovskite, a solution of PDCl (~0.1 mg ml^−1^ in isopropyl alcohol) was spin-coated onto the perovskite surface at 5,000 rpm for 20 s, followed by annealing at 105 °C for another 10 min. The same conditions were used for the deposition of C_60_ and SnO_2_ as above. Finally, the 90 nm ITO transparent electrode was deposited by sputter-coating through a metal mask with an area of 1.1 × 1.1 cm^2^ or 4.1 × 4.1 cm^2^ with a 35-W radiofrequency power and Ar at 1.5 mTorr using the AJA International sputtering system. To complete triple-junction cell fabrication, the silver grid was deposited by thermal evaporation to a thickness of 230 nm and 720 nm, for 1 cm^2^ and 16 cm^2^, respectively, through a mask. Finally, the front of the cell was deposited with 100 nm MgF_2_ for antireflection.

For process optimizations, 1.91-eV perovskite single-junction opaque cells and perovskite–perovskite double-junction semitransparent test cells were also fabricated on glass substrates.

The patterned ITO-coated glass was first prepared by ultrasonic cleaning in deionized water containing 2% Hellmanex, followed by rinses in deionized water, acetone and isopropanol, each for 15 min. The cleaned ITO substrates were then subjected to UVO treatment for 15 min. After UVO treatment, the substrates were transferred to a nitrogen-filled glovebox for subsequent perovskite or perovskite–perovskite test solar cell fabrication.

The 1.91-eV perovskite single-junction opaque solar cell has the structure: glass/ITO/MeO-2PACz/Cs_0.16_Rb_0.04_FA_0.8_Pb(I_0.45_Br_0.55_)_3_/(PDCl)/C_60_/BCP/Cu.

To evaluate the operational stability of Rb and MA incorporation in perovskites, three different compositions were used in fabrication test cells with the structure glass/ITO/MeO-2PACz/perovskite/C_60_/BCP/Cu. The three compositions were:

1. Cs_0.16_Rb_0.04_FA_0.8_Pb(I_0.45_Br_0.55_)_3_ (CsFARb);
2. Cs_0.16_MA_0.04_FA_0.8_Pb(I_0.45_Br_0.55_)_3_ (CsFAMA);
3. Cs_0.2_FA_0.8_Pb(I_0.45_Br_0.55_)_3_ (CsFA).

The same conditions were used as for the 1.55-eV middle perovskite cell for the deposition of MeO-2PACz. The same conditions were also used as above for the deposition of 1.91-eV perovskite, PDCl treatment and C_60_. Instead of SnO_2_, 7 nm BCP was deposited via thermal evaporation. Finally, 100-nm-thick copper electrodes were deposited through a metal mask (to a defined device aperture area of 0.0706 cm^2^) by thermal evaporation, to finish the single-junction perovskite solar cell fabrication.

The 1.55-eV perovskite–1.91-eV perovskite semitransparent tandem test cells were fabricated using the same conditions as those used for triple-junction cells, except the Si bottom cell was replaced by ITO-patterned glass and the cell aperture area was 0.09 cm^2^ and no metal grid was deposited. Electrical contact was made directly to the ITO.

### Device encapsulation

For stability tests, 1-cm^2^ tandem devices were laminated between two pieces of 3-mm-thick glass laminated by transparent polyolefin-type material, with polyisobutylene applied at the edges for sealing^7^. The laminating process was carried out in a Radiant YDS-1111 laminator at 110 °C for 10 min at 800 millibars of pressure. Copper tape was employed to establish electrical contact with the device electrodes, extending outward from the cover glass.

### Characterizations

*J–V* measurements for single-junction opaque devices and perovskite–perovskite semitransparent double-junction devices were performed using a 1 lamp solar cell *I–V* testing system using a class AAA solar simulator under an illumination power of 100 mW cm^−2^. The light was calibrated using a certified reference cell. A scan rate of 100 mV s^−1^ was used during measuring, sweeping from near-open circuit voltage (*V*_OC_) (1.4 V for single junctions, 2.3 V for perovskite–perovskite tandems) to near- short circuit current density (*J*_SC_) (−0.1 V). Apertures of 0.0706 cm^2^ and 0.09 cm^2^ were used for single-junction opaque cells (illuminated from the glass side) and semitransparent double-junction cells (illuminated from the low-bandgap side), respectively.

*J–V* measurements for 1-cm^2^ triple-junction devices were performed using an LED solar simulator (6,060 A, 350–1,200 nm, AAA class, Qingdao Solar Science Instrument Hightech) under an illumination power of 100 mW cm^−2^. A scan rate of 100 mV s^−1^ was used during measurement, sweeping from 3.2 V to near *J*_SC_ (−0.1 V). An aperture of 1.0 cm^2^ was used. *J–V* measurements for 16 cm^2^ triple-junction devices were performed by the Shanghai Institute of Microsystem and Information Technology, Chinese Academy of Science, using a dual light source AAA steady-state solar simulator (YSS-T155A-2M) under an illumination power of 100 mW cm^−2^ with an aperture of 16.0 cm^2^.

EQE measurements for single-junction solar cells were carried out using the QuantX-300 Spectral Response (Newport) system with monochromatic light from a xenon arc lamp.

EQE measurements for triple-junction tandem solar cells were carried out in AC mode using Enli Technology (model QE-R) Taiwan system. The EQE response was calibrated using a certified reference cell for 300–1,100 nm. For measuring EQE of the silicon bottom cell, a blue LED (450 nm) and infrared LED (730 nm) were used to saturate the top and the middle cells. For measuring EQE of the middle perovskite cell, a blue LED (450 nm) and near-infrared LED (940 nm) were used to saturate the top and the bottom cells. For measuring EQE of the top perovskite cell, an infrared LED (730 nm) and near-infrared LED (940 nm) were used to saturate the middle and the bottom cells.

Transient photocurrents of solar cells were measured using a Keysight MSO9254A oscilloscope. The 520 nm wavelength excitation light was provided by a Thorlabs NPL52B pulsed laser with a 5-ns pulse width, repetition rate of 1 MHz and pulse energy of 1.2 nJ. The diameter of the beam was approximately 2 mm.

Temperature-dependent open circuit voltages of solar cells were measured using a Keysight 2636B source meter, illuminated by a Thorlabs OSL2 Fiber-Coupled Illuminator with intensity equivalent to 1 Sun. The temperature was controlled using a cryogenic cryogen-free variable temperature cryostat, with a Lakeshore 350 temperature controller.

The transmittance and reflectance of samples were measured using a Perkin Elmer Lambda1050 UV/Vis/NIR spectrophotometer.

Absorbances of samples were measured using an FS 5 (Edinburgh Instruments).

Thermal admittance spectroscopy and Mott–Schottky were conducted using a Keysight E4990A impedance analyser, operating from 20 Hz to 10 MHz with the ‘enhanced measurement speed’ option.

Time-of-flight secondary ion mass spectrometry (TOF-SIMS) was performed using an IONTOF TOF-SIMS 5 system, operating in positive polarity mode with Bi^3+^ primary ions at an energy of 30 keV and Cs^+^ sputtering ions at 1 keV, in the MC^s+^ operational mode

X-ray diffraction patterns were recorded using a Bruker ECO D8 diffractometer with a Cu*K*α (*λ* = 1.5418 Å) radiation.

XPS and UPS were performed using an ESCALAB250Xi (Thermo Scientific). For XPS analysis, we employed X-ray emission using an anode with Mg*K*α line (12 kV–200 W) from an ultrahigh vacuum non-monochromatic source. Following an initial survey scan to assess chemical states, we performed high-resolution scans at a pass energy of 10 eV. The excitation energy used was 1,253.6 eV. The *Φ* was calculated according to the formula *Φ* = *hν* (21.22 eV) − *E*_cutoff-measured_.

Top-view and cross-sectional SEM images were obtained using a field-emission microscope (NanoSEM 230).

### TEM including specimen preparation

To characterize gold Au deposited (for different durations) on the ALD SnO_2_ layer, a carbon-coated TEM grid (EMSCF200-CU-UL, Proscitech) was used, which was coated with a 20-nm SnO_2_ layer by ALD, followed by thermal evaporation of Au for different times.

The samples were introduced into a JEOL 2100F FEG-TEM, which was fitted with a Gatan Ultrascan camera for imaging. For the TEM imaging process, we applied an electron dose rate of approximately 2 e/Å² per second.

### Photoluminescence characterizations

Steady-state photoluminescence spectra of perovskite films were measured using an FS 5 (Edinburgh Instruments) with an excitation wavelength of 450 nm.

For TRPL decay measurements, a LabRAM HR Evolution system was used with a time-correlated single photon counting system (DeltaPro-DD, Horiba). Using a 485-nm diode laser (DD-510L, Horiba) as the excitation source, a laser with a pulse duration of 110 picoseconds, a reception rate of 312.5 kilohertz and a fluence of approximately 5–6 microjoules per square centimetre per pulse was used. The PL signal was captured at a wavelength of 660 nm. Both the incident and reflected light were directed through a ×50 objective lens (Leica PL FLUOTAR L 50/0.55), resulting in a spot size of approximately 2 μm. The samples were maintained in a nitrogen environment to prevent degradation during the measurement process. To determine the PL lifetime from the TRPL decay curves, a bi-exponential model was applied:

$$y={A}_{1}{\mathrm{{e}}}^{-\frac{t}{{\tau }_{1}}}+{A}_{2}{\mathrm{{e}}}^{-\frac{t}{{\tau }_{2}}}$$

where *A*_1_ and *A*_2_ are weightings of the *τ*_1_-fast decay component recombination via defect trapping and of the *τ*_2_-slow decay component associated with radiative recombination^15^ was used in decay analysis software to fit the experimental results. The average lifetime, *τ*_avg_ was calculated using the following equation:

$${{\tau}}_{{\rm{avg}}}=\frac{{A}_{1}\times {\tau }_{1}^{2}+{A}_{2}\times {\tau }_{2}^{2}}{{A}_{1}\times {\tau }_{1}+{A}_{2}\times {\tau }_{2}}$$

For PL imaging, a custom PL imaging system, featuring 430-nm royal-blue LED chips and 451/106-nm bandpass filters, was employed. The cells were secured in a nitrogen-filled, temperature-controlled custom jig during the imaging process and exposed to an intensity equivalent to 1 Sun. To capture the PL image, a Peltier-cooled (at −70 °C) Si CCD camera from Princeton Instruments (model Pixis 1024) along with a 700-nm long-pass filter was used, with an exposure time of 0.03 s. The PL image was then processed using Fiji software, which was also used to add a colour bar and calibration bar to the image.

### Stability testing

For MPPT of the solar cells to evaluate operational stability of triple-junction tandems, encapsulated devices were placed inside an environmental chamber for continuous Xe illumination (100 mW cm^−2^). The temperature and relative humidity were kept at 25 ± 5°C and 60 ± 20%, respectively. The MPPT algorithm is based on the well-established perturb-and-observe methodology, integrated into a LabVIEW program for efficient implementation. The algorithm begins by deriving an initial estimation of the MPP through a rapid initial *J–V* measurement. In the regular operation of the algorithm, the applied voltage is perturbed using a double step of both +10 mV and −10 mV around the voltage corresponding to the maximum power point, denoted as *V*_MPP_. Subsequently, the output power of the solar cell is measured at these three distinct voltage levels. The algorithm then selects the new *V*_MPP_ on the basis of the voltage configuration that yields the highest power output. One critical aspect of the algorithm’s execution is the duration of each voltage step. It is imperative to ensure that this duration is sufficiently long to allow for transients within the system to equilibrate before computing the power at the newly set voltage level. This careful consideration ensures the accuracy and effectiveness of the MPPT process.

For IEC 61215 standard thermal cycling testing, the encapsulated triple-junction tandem device underwent a thermal cycling regime and was measured ex situ regularly. The temperature cycle was between −40 °C and 85 °C, and for 204 times, in this work. During the cyclic testing, the device was held at both −40 °C and 85 °C for a duration of 10 min each. The temperature transitions between these points were executed at a controlled ramp rate of 45 °C per hour.

### Simulation

Simulation of the electrostatic surface potential of PD^+^ was carried out by the DFT/B3LYP method with a basis set of 6-31G(d)(p) for determining the dipole moment. All the calculations were performed using the Gaussian 16 program package.

A commercial software package, Silvaco technology computer-aided design, was used to model the energy band structure of the SnO_2_/(Au)/NiO_*x*_ stack under thermal equilibrium.

To calculate the optical effect of the Au nanoparticles, the Python-based software RayFlare^28^ was used, which uses a modified version of the solver S^4^ (ref. ^29^). The nanoparticles were represented as Au pillars in an NiO_*x*_ background material. It is assumed that the nominal thickness of Au (*d*_Au,nominal_) deposited can be used to calculate the total volume of Au deposited per unit area, so that the height of the Au pillars (*h*_pillar_) can be calculated from *h*_pillar_ = *d*_Au,nominal_/*C*, where *C* is the area coverage fraction of the Au. The coverage fraction was determined from scanning TEM images of Au deposited at different nominal thicknesses. Since RCWA calculations assume a periodic unit cell, the random structure of the Au nanoparticles was simulated by generating a random unit cell, containing eight non-overlapping Au pillars randomly placed within the unit cell. The radius of the discs and size of the unit cell were chosen to give the correct coverage fraction with the pillar height as calculated above. The cell structure used is shown in Fig. 3a. Twenty random unit cells were generated for each coverage fraction, with the reflectance, transmittance and absorptance per layer calculated for each unique unit cell assuming normally incident unpolarized light. We then took the average of these results. The maximum possible short circuit current of the two perovskite junctions was calculated as:

$${J}_{\max ,i}=q{\int }_{280\;{\rm{nm}}}^{1,200\;{\rm{nm}}}{\Phi }_{{\mathrm{AM}}1.5{\mathrm{G}}}(\lambda )A_{i}(\lambda ){\mathrm{d}}\lambda$$

where *Φ*_AM1.5G_(*λ*) is the photon flux in the AM1.5G solar spectrum as a function of wavelength, *q* is the elementary charge and A_*i*_(*λ*) is the fraction of incident photons absorbed in the relevant perovskite layer, from the average of the 20 randomly generated unit cells. For the results without antireflection coating, the simulations were performed using RCWA only. To simulate cells with textured polydimethylsiloxane antireflection coating, RayFlare’s ray tracer was used, assuming a regular inverted pyramid structure with an opening angle of 52°.

Simulation codes for results in Supplementary Figs. 18–21 can be found in ref. ^30^.

### Reporting summary

Further information on research design is available in the Nature Portfolio Reporting Summary linked to this article.

## Online content

Any methods, additional references, Nature Portfolio reporting summaries, source data, extended data, supplementary information, acknowledgements, peer review information; details of author contributions and competing interests; and statements of data and code availability are available at 10.1038/s41565-025-02015-x.

## Supplementary information

Supplementary Information Supplementary Figs. 1–28, Tables 1–5 and refs.

Reporting Summary

## Data Availability

The data supporting the findings of this study and the minimum dataset for the interpretation, verification and extension of the research are available in the Article and Supplementary Information. Additional data are available from the corresponding authors on reasonable request.

## Code availability

The optical simulation code is available via Zenodo at 10.5281/zenodo.16215154 (ref. ^30^).
