# Supplementary material for: Tailoring nanoscale interfaces for perovskite–perovskite–silicon triple-junction solar cells
Source: Nat Nanotechnol. 2025 Oct 7;20(11):1648–55. doi: 10.1038/s41565-025-02015-x (PMC12623231; doi:10.1038/s41565-025-02015-x)
Supplement: Supplementary file 1 — Supplementary Figs. 1–28, Tables 1–5 and refs. [file 41565_2025_2015_MOESM1_ESM.pdf]

nature nanotechnology

Supplementary information https://doi.org/10.1038/s41565-025-02015-x

Tailoring nanoscale interfaces for perovskite–perovskite–silicon triple-junction solar cells

In the format provided by the authors and unedited

Table of contents:

Supplementary figures 1 to 28

Supplementary tables 1 to 5

Supplementary reference 1 to 9

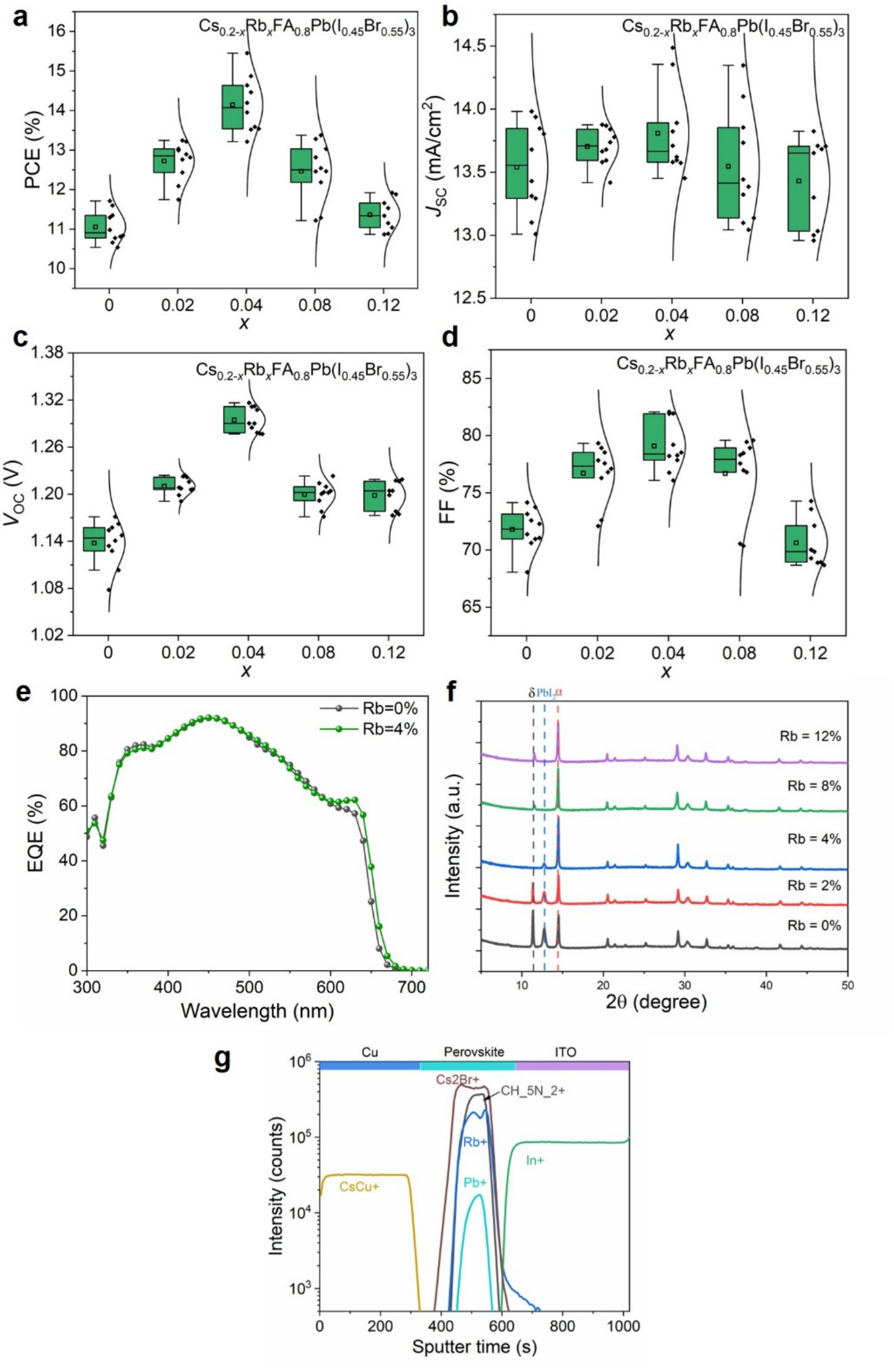

Supplementary figure 1 Effect of varying Rb in $Cs_{0.2-X}Rb_XFA_{0.8}Pb(I_{0.45}Br_{0.55})_3$ high bandgap perovskite solar cell performance, absorption threshold and perovskite film crystallinity and elemental distribution. Distributions of (a) PCE， (b) J_{SC}, (c) V_{OC} and (d) FF (10 devices in each type. Top value: maximum; top bar: 75th percentile; middle bar: median; open squares: mean; bottom bar: 25th percentile; lowest value: minimum; solid diamonds: measured data). (e) External quantum efficiencies (EQE) of $CsFA_{0.8}Pb(I_{0.45}Br_{0.55})_3$ (X_{Rb} = 0) and $Cs_{0.16}Rb_{0.04}FA_{0.8}Pb(I_{0.45}Br_{0.55})_3$ device of indicating a reduction in absorption threshold after Rb incorporation. (f) X-ray diffraction (XRD) pattern of $Cs_{0.2-X}Rb_XFA_{0.8}Pb(I_{0.45}Br_{0.55})_3$ perovskite film where X= 0; 0.02; 0.04; 0.08 or

0.12 showing minimum PbI_2 peak when X=0.04. (g) Time-of-flight secondary ion mass spectrometry (TOF-SIMS) of $Cs_{0.16}Rb_{0.04}FA_{0.8}Pb(I_{0.45}Br_{0.55})_3$ perovskite cells.

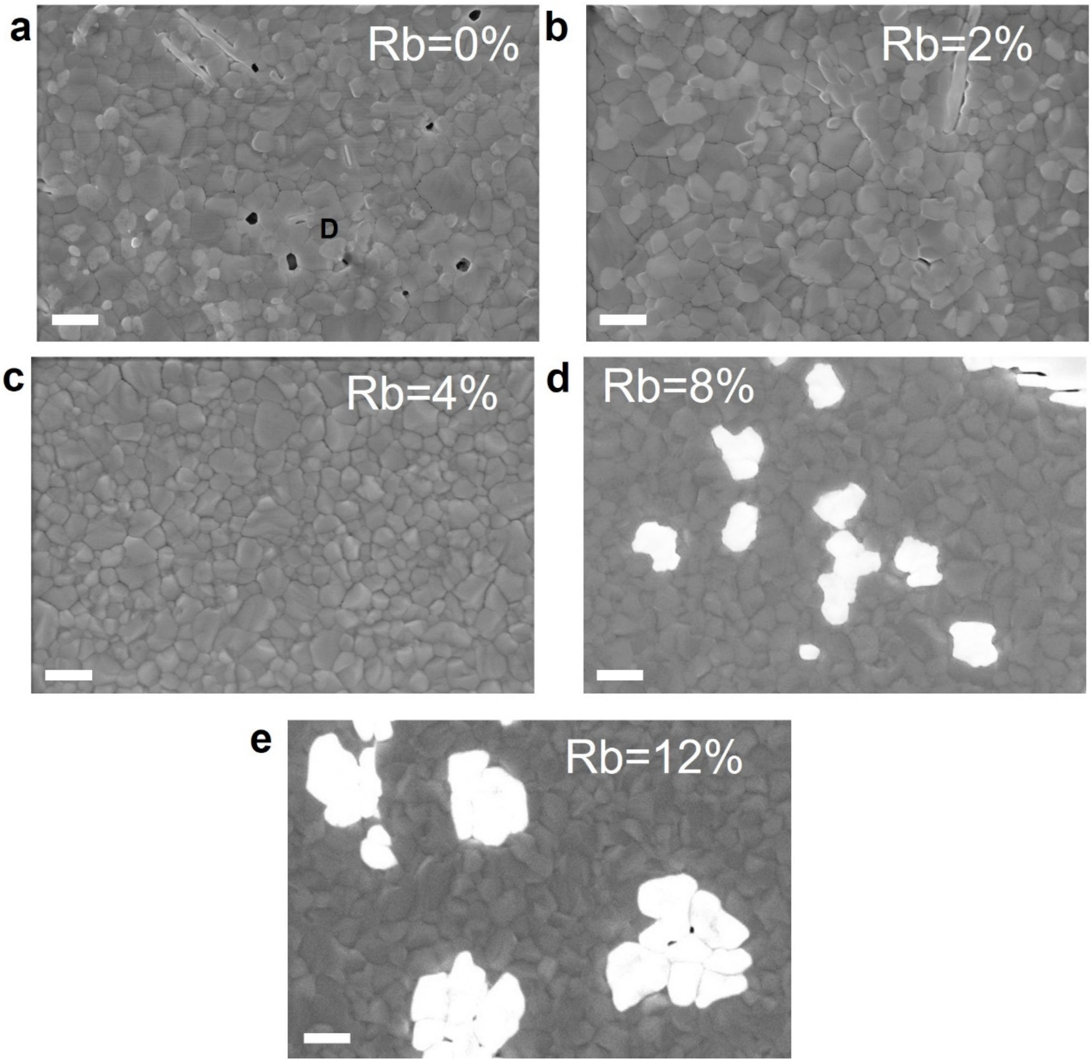

Supplementary figure 2 Scanning Electron Microscopy (SEM) top-view images of high bandgap $Cs_{0.2-X}Rb_XFA_{0.8}Pb(I_{0.45}Br_{0.55})_3$ perovskite film where X= 0; 0.02; 0.04; 0.08 or 0.12. Rb is mostly uniformity distributed when X = 0.04. (scale bar = 400 nm)

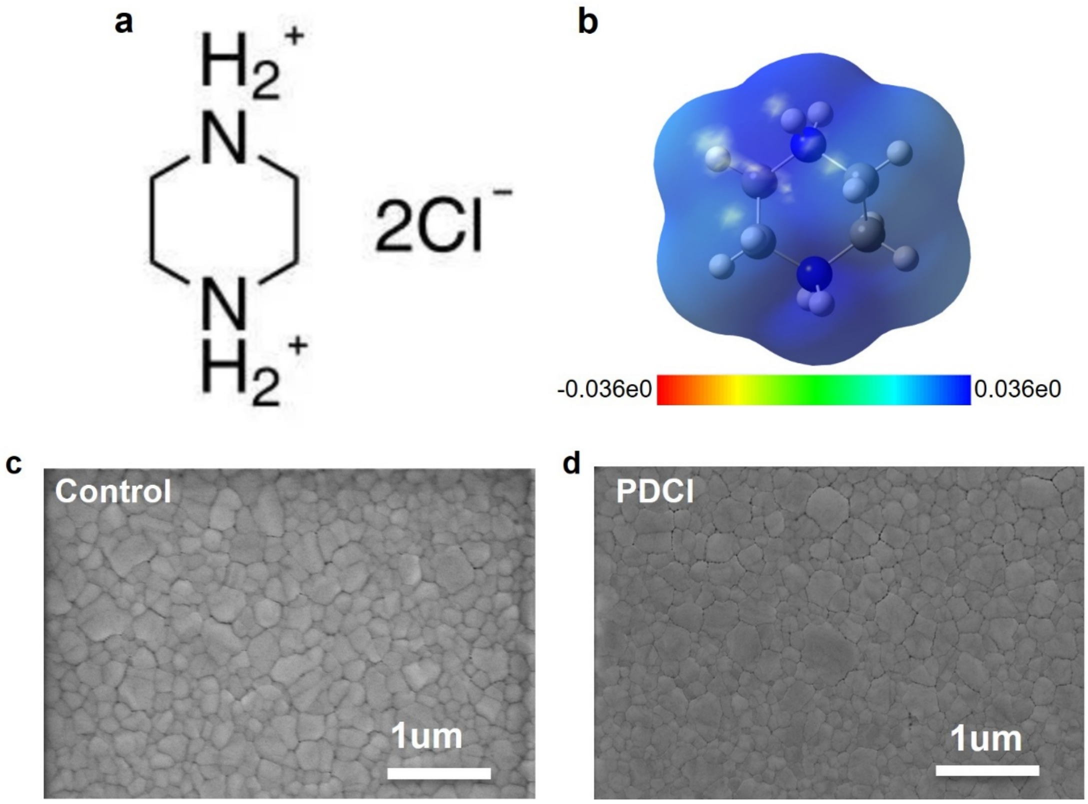

Supplementary figure 3 Piperazine-1,4-diium chloride (PDCl) for surface treatment. (a) Molecular structure of PDCl and (b) electrostatic potential (ESP) of PD^+. Top view scanning electron microscopy (SEM) of (c) control (untreated) and (d) PDCl treated $Cs_{0.16}Rb_{0.04}FA_{0.8}Pb(I_{0.45}Br_{0.55})_3$ perovskites.

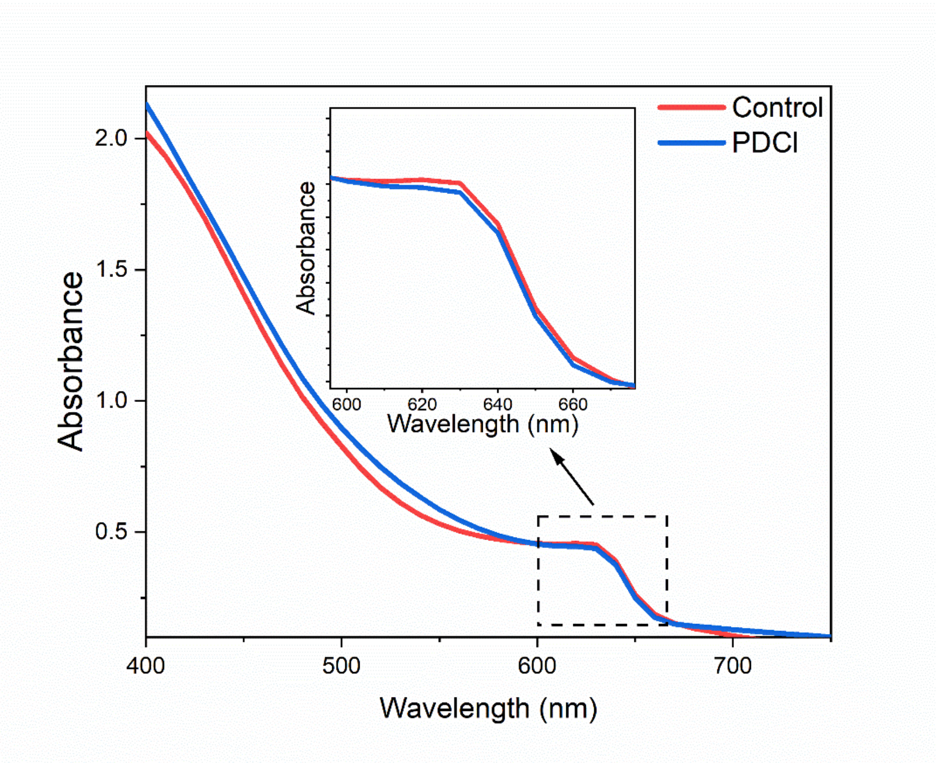

Supplementary figure 4 Absorbance of $Cs_{0.16}Rb_{0.04}FA_{0.8}Pb(I_{0.45}Br_{0.55})_3$ perovskite film without (red) and with (blue) PDCl surface treatment.

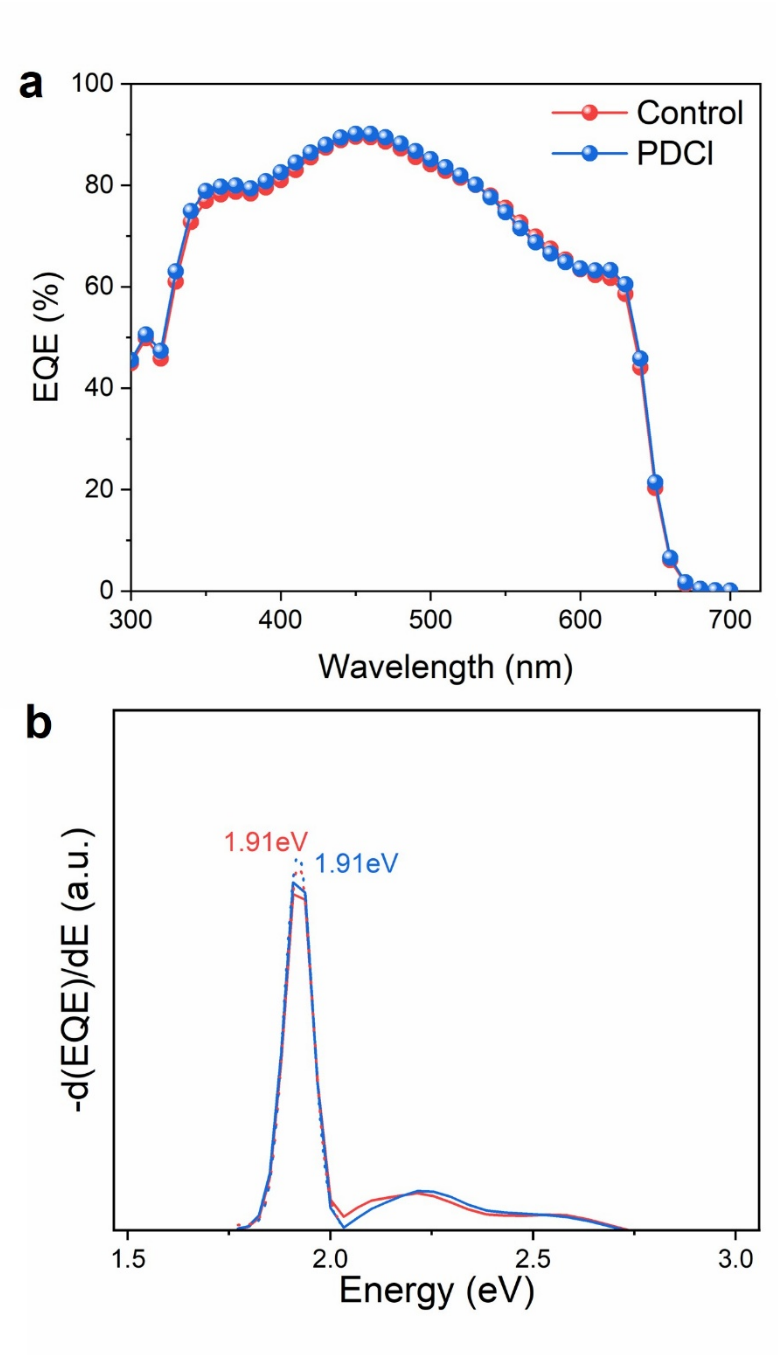

Supplementary figure 5 (a) EQE of representative and (b) calculated absorption threshold (Eth (loosely defined as Eg)) of $Cs_{0.16}Rb_{0.04}FA_{0.8}Pb(I_{0.45}Br_{0.55})_3$ cells without (red) and with (blue) PDCl treatment.

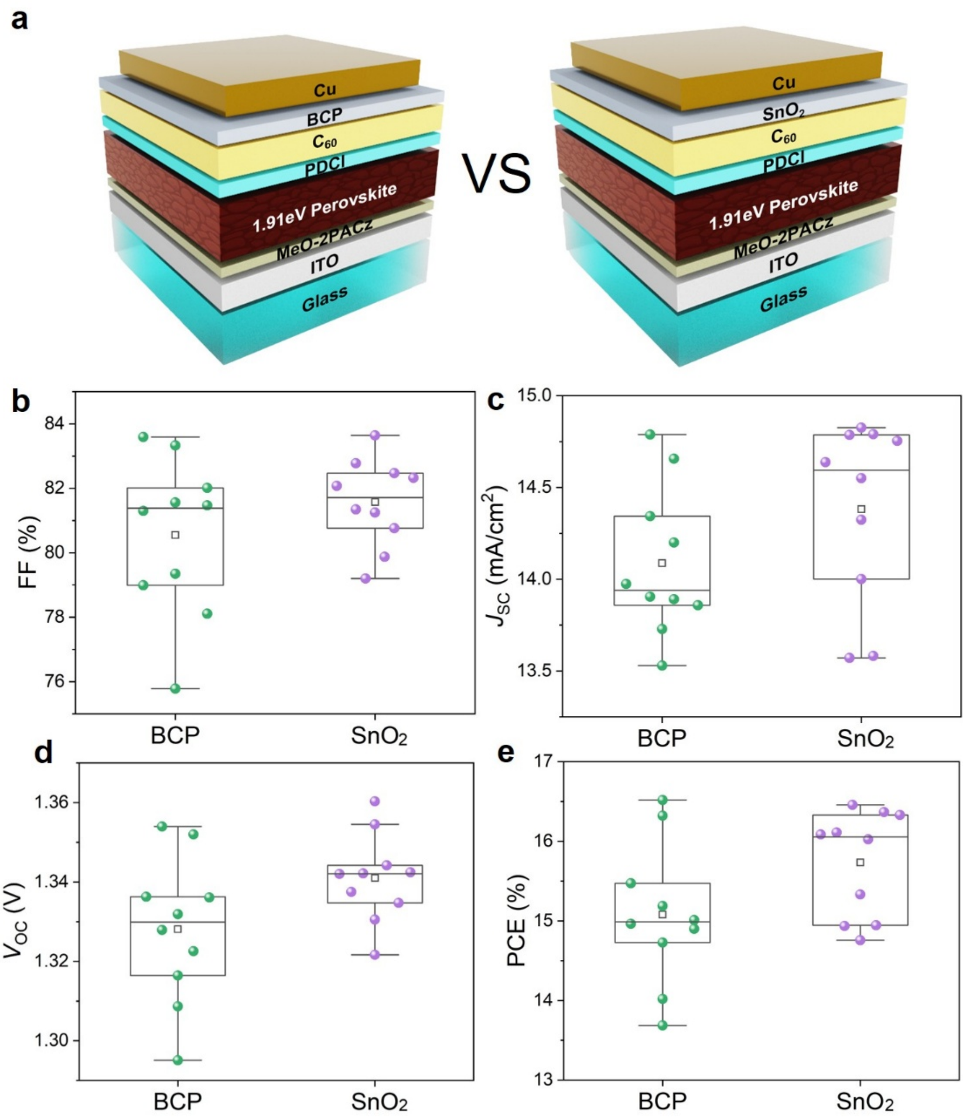

Supplementary figure 6 (a) Schematic of BCP- (left) vs SnO_2– (right) based high bandgap (1.91eV) perovskite p-i-n devices and their distributions of (b) FF, (c) J_{SC}, (d) V_{OC} and (e) PCE of PDCl treated (10 devices in each type. Top value: maximum; top bar: 75th percentile; middle bar: median; open squares: mean; bottom bar: 25th percentile; lowest value: minimum; solid circles: measured data).

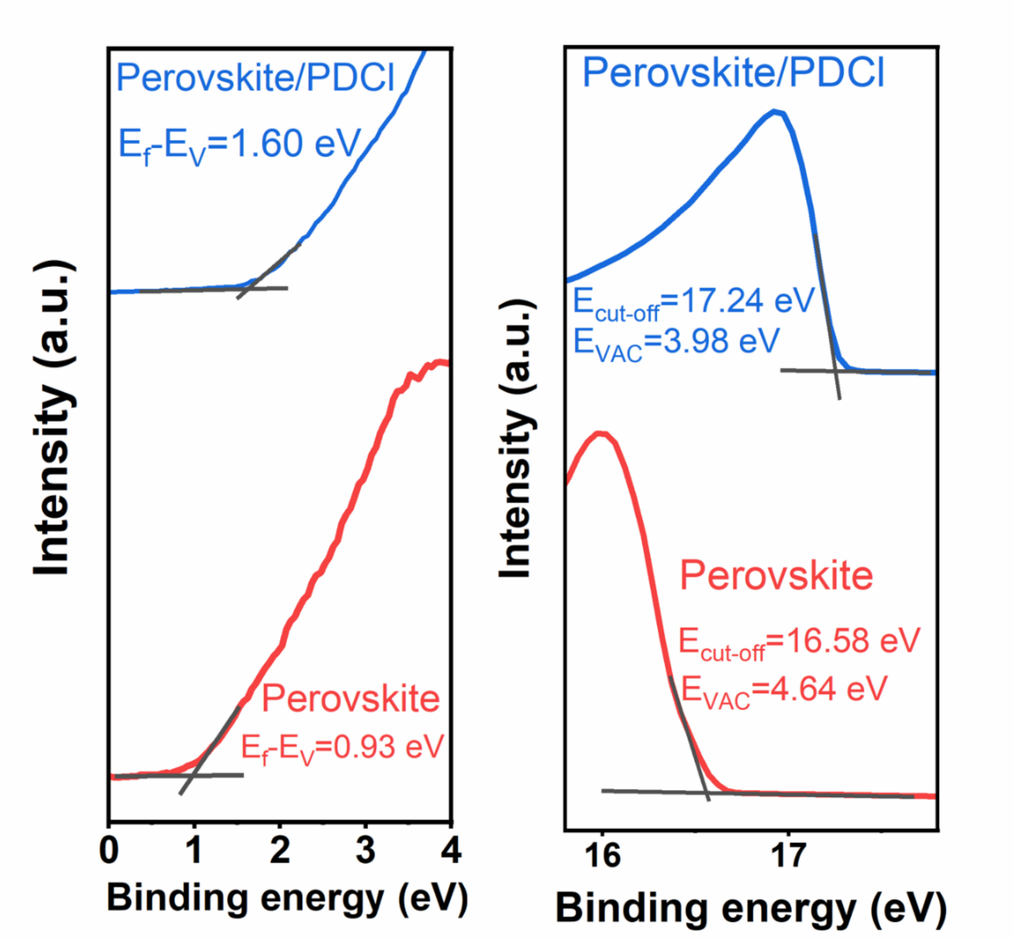

Supplementary figure 7 Ultraviolet photoelectron spectroscopy (UPS). UPS spectra result of control (untreated) and PDCl-treated (1.91eV) $Cs_{0.16}Rb_{0.04}FA_{0.8}Pb(I_{0.45}Br_{0.55})_3$ perovskites showing corresponding vacuum level (E_{VAC}) determined by hν (21.22 eV) – E_{cutoff} measured on the valence band maxima (E_V) of the perovskites relative to the Fermi level (E_F) on the right. E_g are determined from **Supplementary figure 5B**.

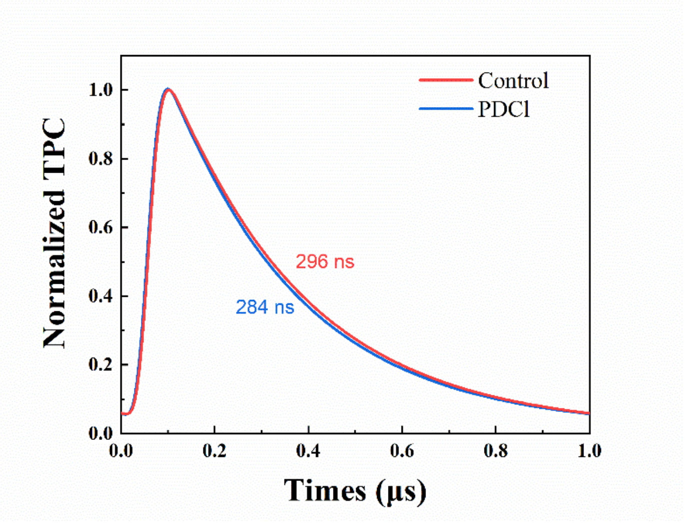

Supplementary figure 8 Transient photocurrent (TPC) measurements of 1.91eV $Cs_{0.16}Rb_{0.04}FA_{0.8}Pb(I_{0.45}Br_{0.55})_3$ cells without (red) and with (blue) PDCl treatment.

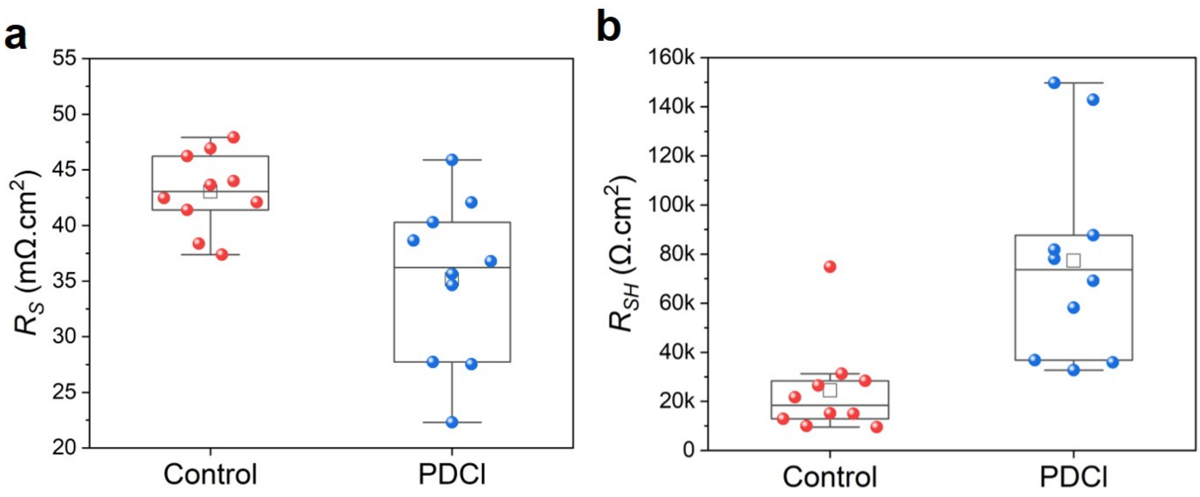

Supplementary figure 9 Distributions of (a) R_S and (b) R_{SH} of 1.91eV $Cs_{0.16}Rb_{0.04}FA_{0.8}Pb(I_{0.45}Br_{0.55})_3$ cells without (red) and with PDCl treatment (blue). (10 devices in each type. Top value: maximum; top bar: 75th percentile; middle bar: median; open squares: mean; bottom bar: 25th percentile; lowest value: minimum; solid circles: measured data)

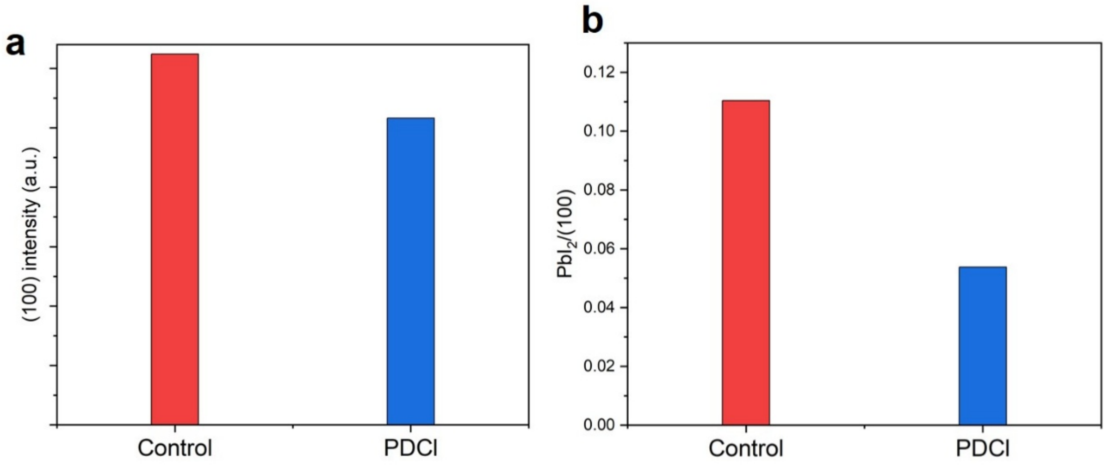

Supplementary figure 10 (a) Intensity of the (100) XRD and (b) intensity ratios of PbI_2/(100) XRD peaks of 1.91eV perovskite films from without (red) and with (blue) PDCl treatment **Figure 2B**.

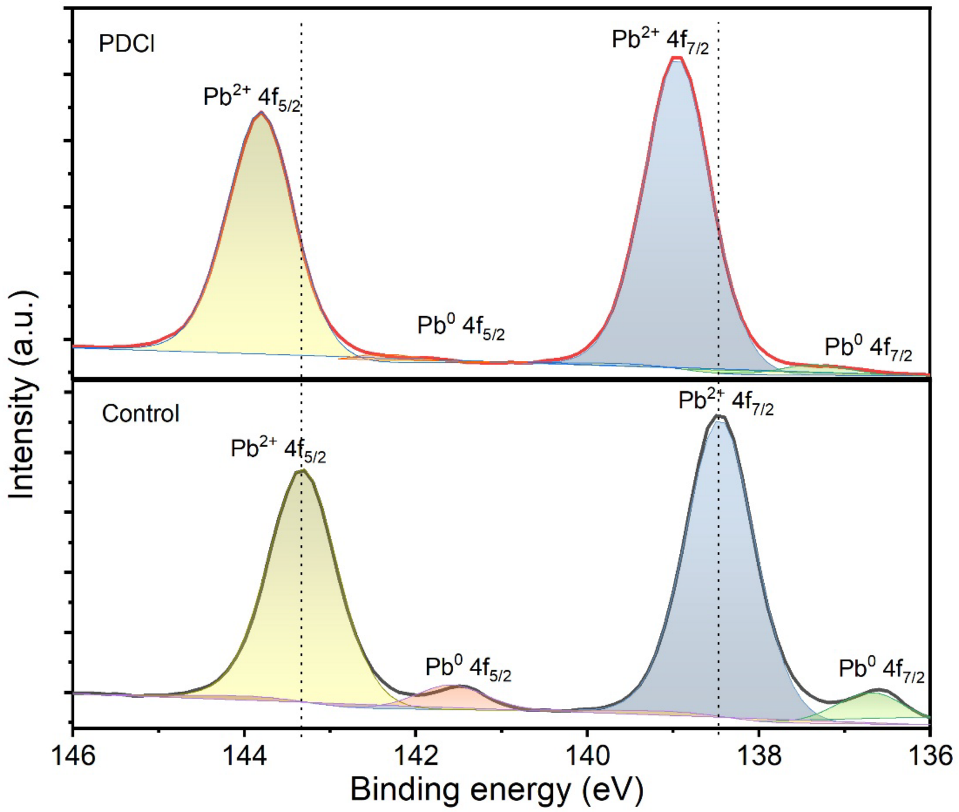

Supplementary figure 11 X-ray photoelectron spectroscopy (XPS). XPS spectra of Pb 4f for control (untreated) and PDCl-treated 1.91 eV $Cs_{0.16}Rb_{0.04}FA_{0.8}Pb(I_{0.45}Br_{0.55})_3$ perovskite films.

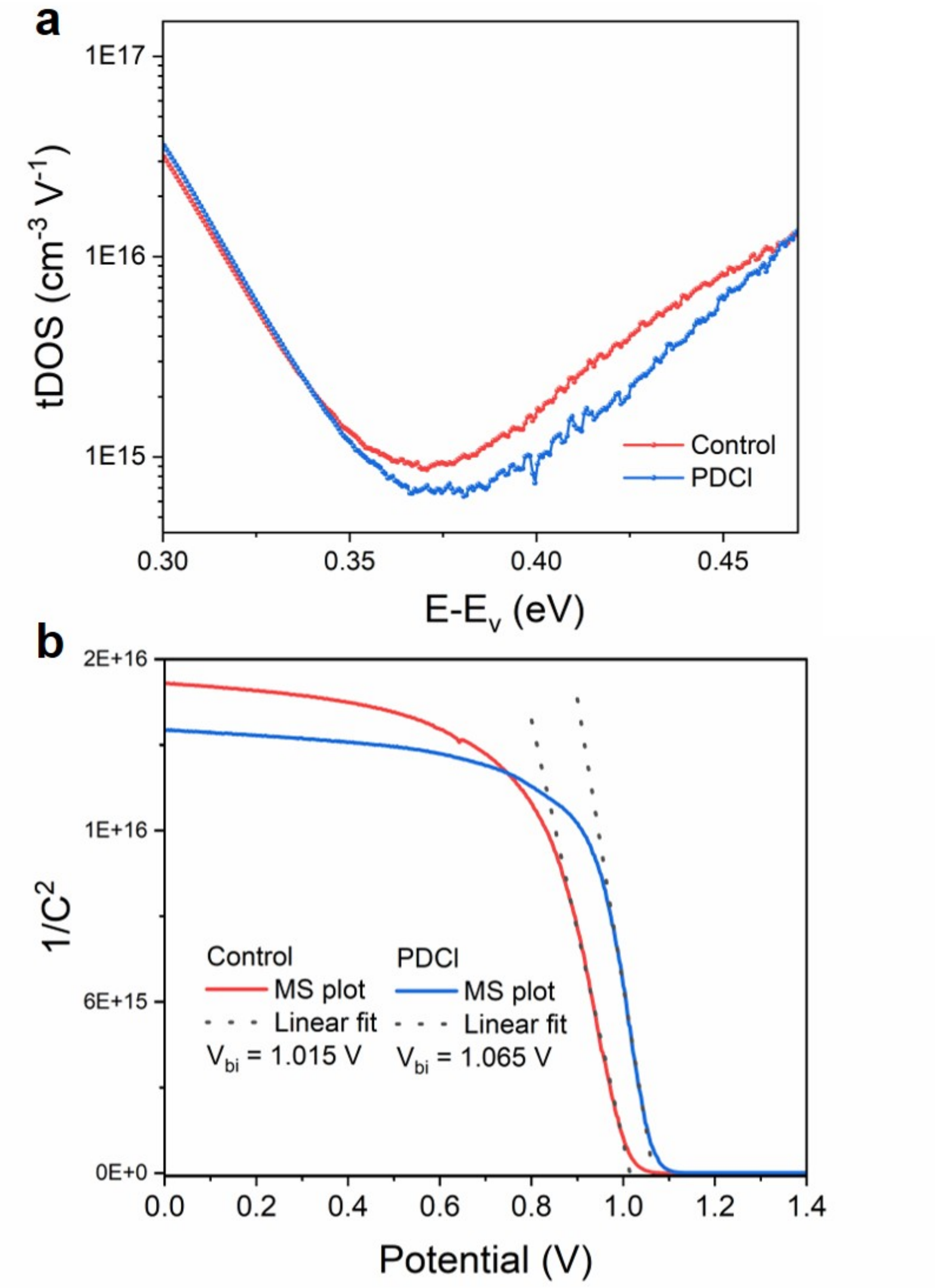

Supplementary figure 12 Defect analyses of 1.91eV $Cs_{0.16}Rb_{0.04}FA_{0.8}Pb(I_{0.45}Br_{0.55})_3$ perovskite solar cells (a) Density of defect states determined by thermal admittance spectroscopy (TAS) of control (untreated) and PDCl-treated devices and (b) Mott-Schottky plots for the same devices determining the built-in-potentials.

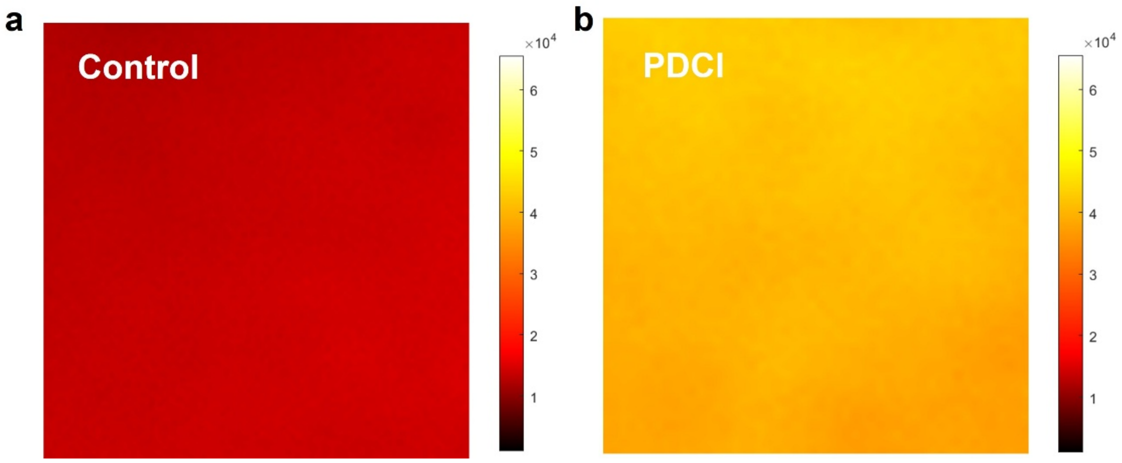

Supplementary figure 13 Photoluminescence images of (a) control (untreated) and (b) PDCl treated 1.91eV perovskites film (image area: 1*1 mm).

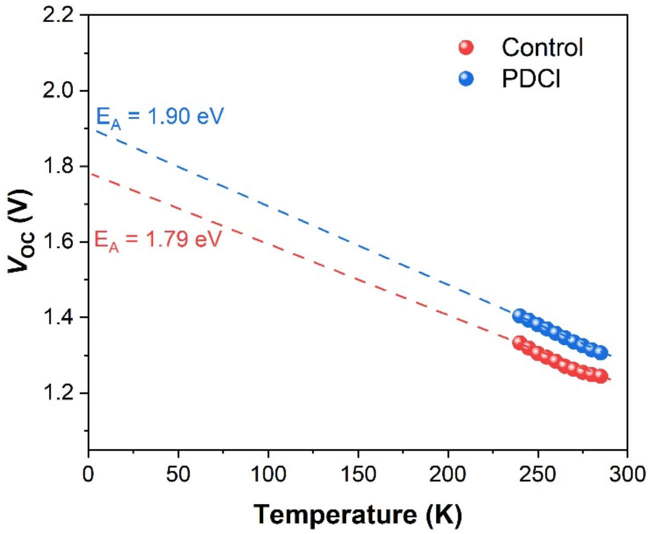

Supplementary figure 14 Temperature-dependent V_{OC} of 1.91eV $Cs_{0.16}Rb_{0.04}FA_{0.8}Pb(I_{0.45}Br_{0.55})_3$ perovskite solar cells.

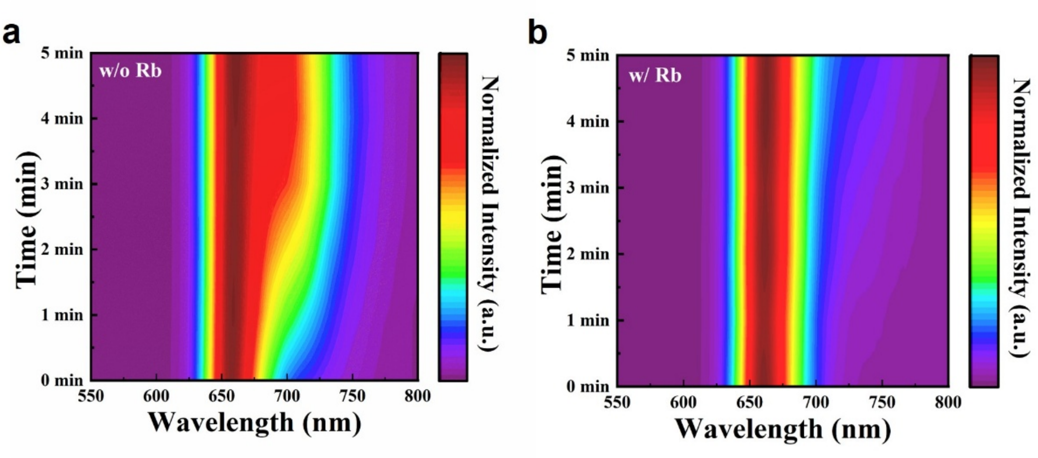

Supplementary figure 15 Time evolution of steady-state PLs for (a) Rb-free $Cs_{0.2}FA_{0.8}Pb(I_{0.45}Br_{0.55})_3$ and (b) $Cs_{0.16}Rb_{0.04}FA_{0.8}Pb(I_{0.45}Br_{0.55})_3$ perovskites.

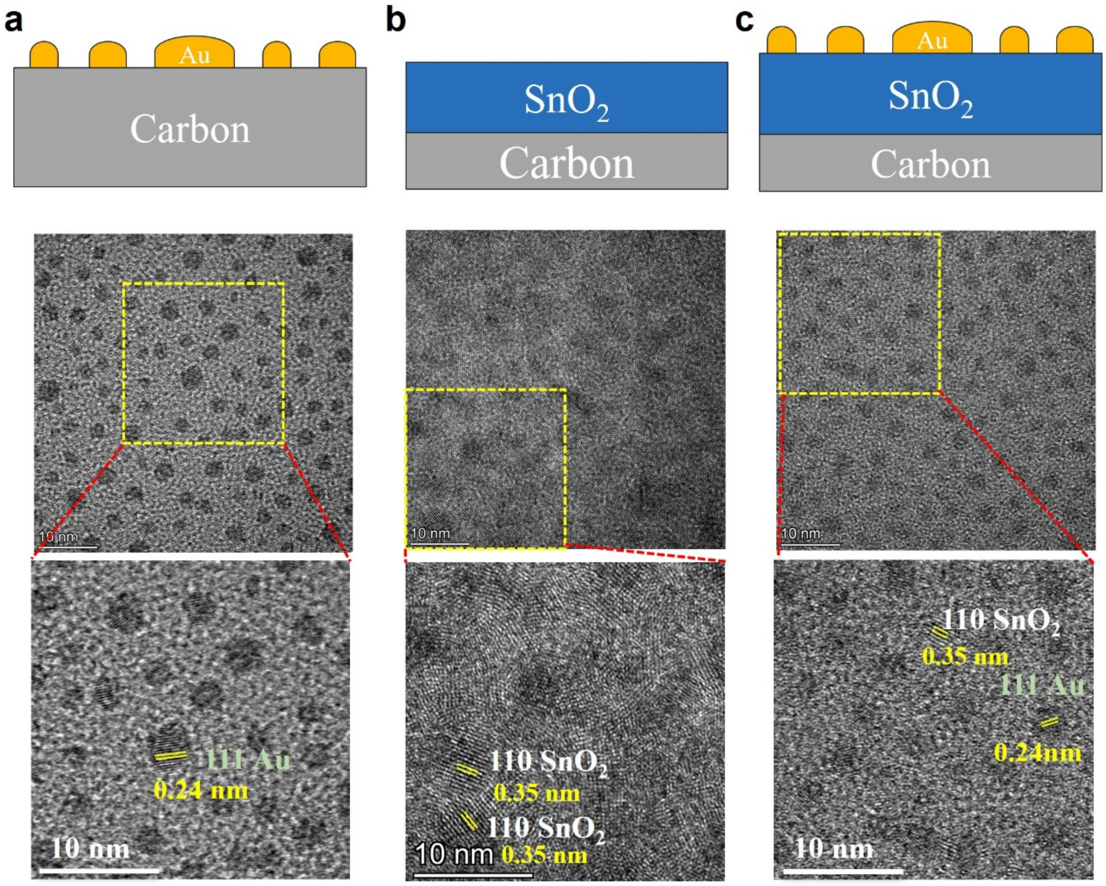

Supplementary figure 16 Transmission electron microscopy (TEM) image of (a) "0.4 nm thick" Au nanoparticle on carbon, (b) 20nm ALD SnO_2 on carbon and (c) "0.4 nm thick" Au nanoparticles on ALD SnO_2/carbon. The identifies of Au and SnO_2 are confirmed and lattice spacings of each material can be seen. Au and SnO_2 dominant diffraction rings are shown in Figure S17.

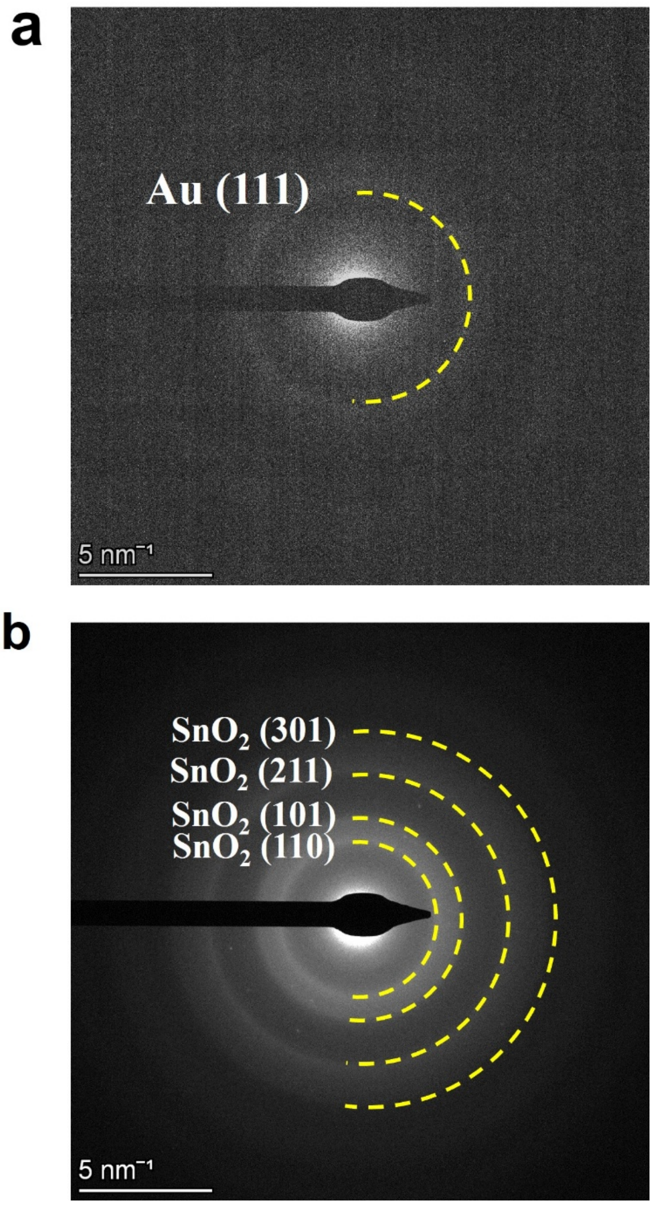

Supplementary figure 17 TEM diffraction rings of (a) Au and (b) SnO_2 on carbon

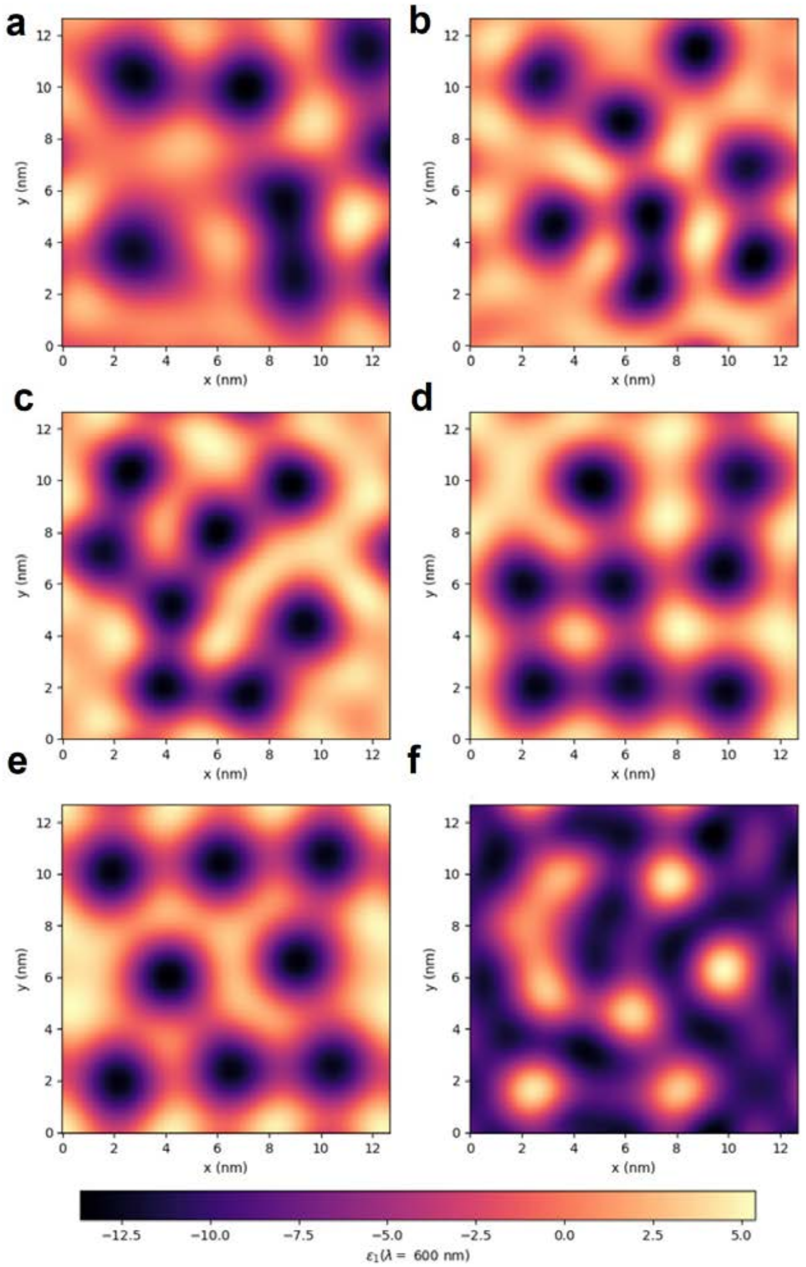

Supplementary figure 18 Representative features for the randomly-generated unit cells used in optical simulation of (a) “0.2”, (b), “0.4”, (c) “0.6”, (d) “0.8”, (e) “1.0”, and (f) “3.0” nm “thick” Au nanoparticles on SnO_2.

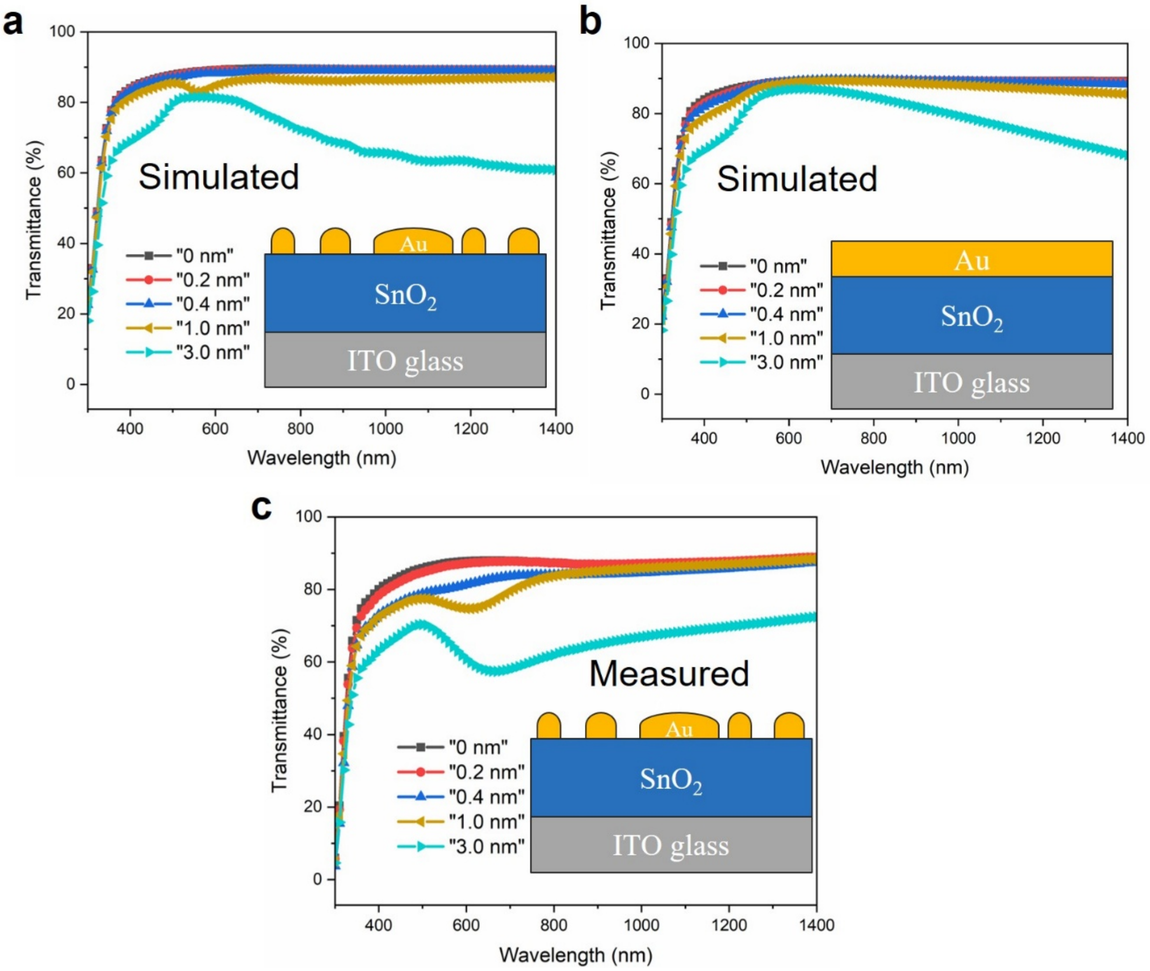

Supplementary figure 19 Simulated transmittance of Au (a) nanoparticles or (b) planar film and (c) measured transmittance of Au nanoparticles on SnO_2 (20 nm)/ITO/glass. The curves are labelled using nominal thickness reading from the thermal evaporator.

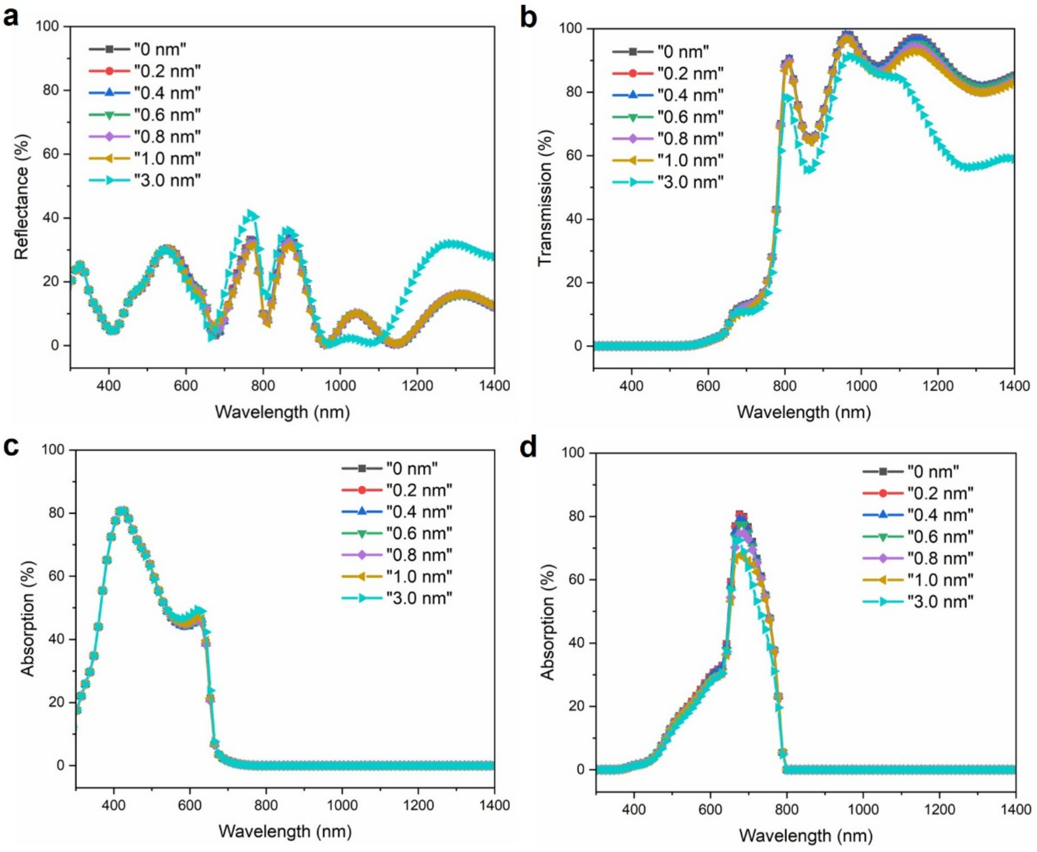

Supplementary figure 20 Optical modelling of a semi-transparent perovskite-perovskite tandem with cell structure shown in **Figure 3a**. (a) Reflectance, (b) transmittance of the entire tandem. (c) Absorption in the high-bandgap perovskite layer. (d) Absorption in the mid-bandgap perovskite layer. There is significant parasitic absorption in the Au nanoparticles, with coverage increasing with nominal thickness, thereby reducing transmittance of the entire tandem in (b) and reducing absorption in the mid-bandgap perovskite layer in (d).

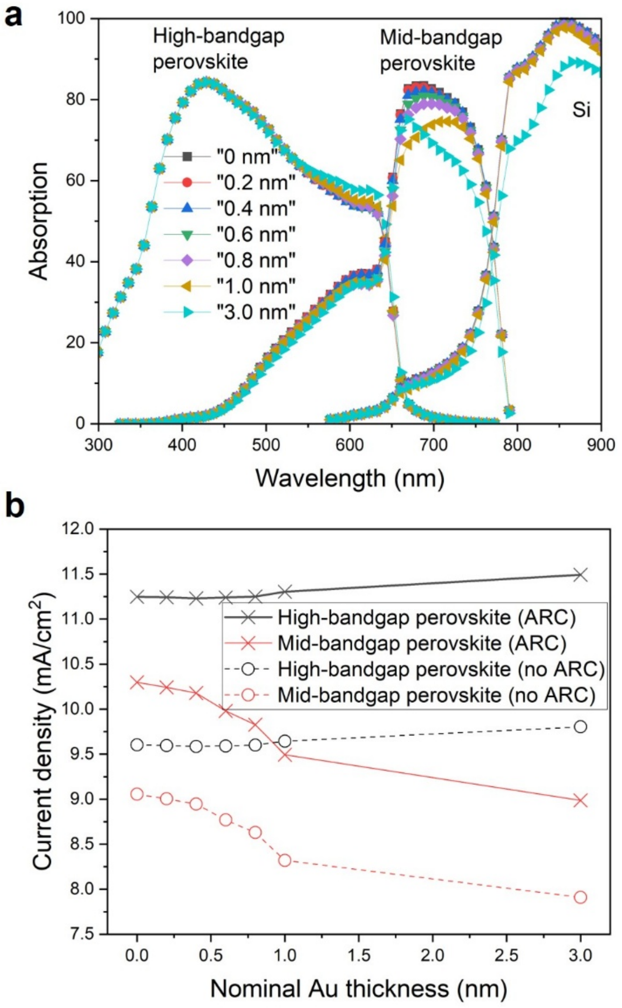

Supplementary figure 21 Simulated (a) absorptance and (b) J_{SC} values (assuming 100% internal quantum efficiency) of perovskite-perovskite-Si tandem (Figure 4a) as a function of nominal Au “thickness” as read out by the thermal evaporator thickness monitor.

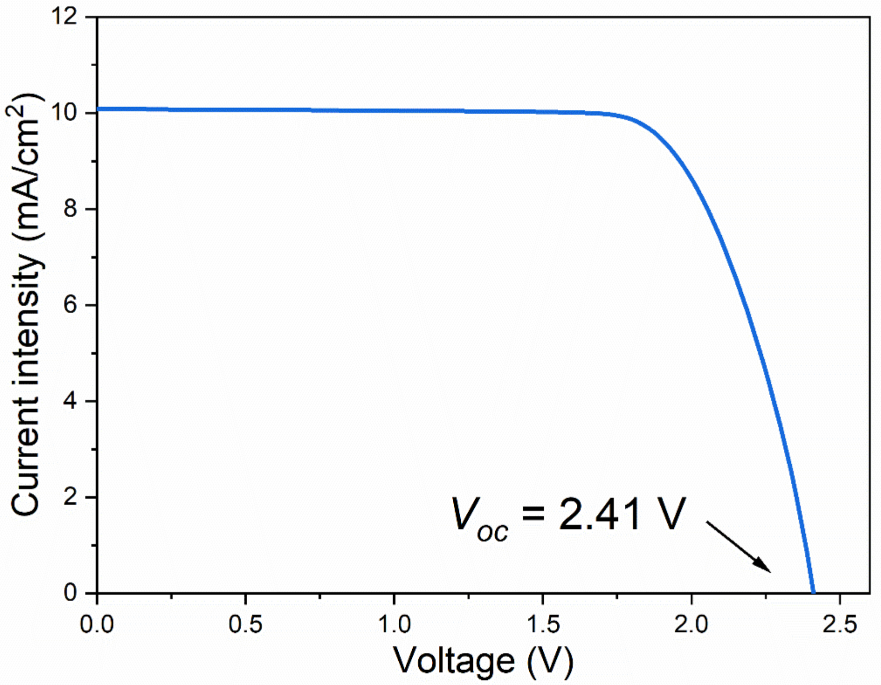

Supplementary figure 22 *J-V* curve of a 1.55eV perovskite - 1.91eV perovskite double junction semitransparent test cell, similar to one shown **Figure 3a**, but after Rb incorporation and PDCl treatment, therefore achieving higher V_{OC} compared to those in **Figure 3b**.

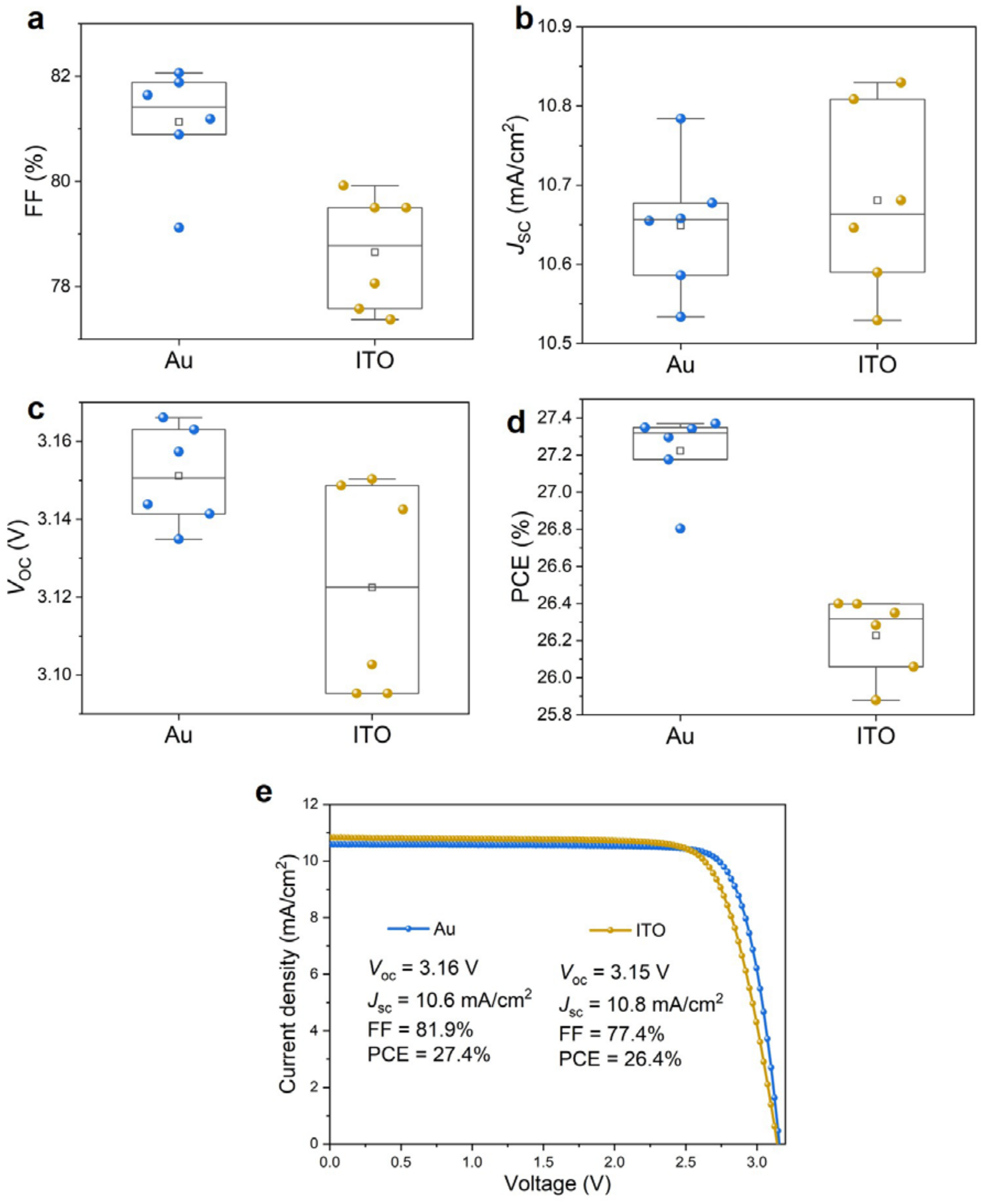

Supplementary figure 23 Distributions of (a) FF, (b) J_{SC}, (c) V_{OC} and (d) PCE of triple junction solar cells with Au nanoparticles or 15nm ITO for interfacing the top and middle perovskite junctions (6 devices in each type. Top value: maximum; top bar: 75th percentile; middle bar: median; open squares: mean; bottom bar: 25th percentile; lowest value: minimum; solid circles: measured data). (e) Reverse scan *J-V* curves of the champion device in each group.

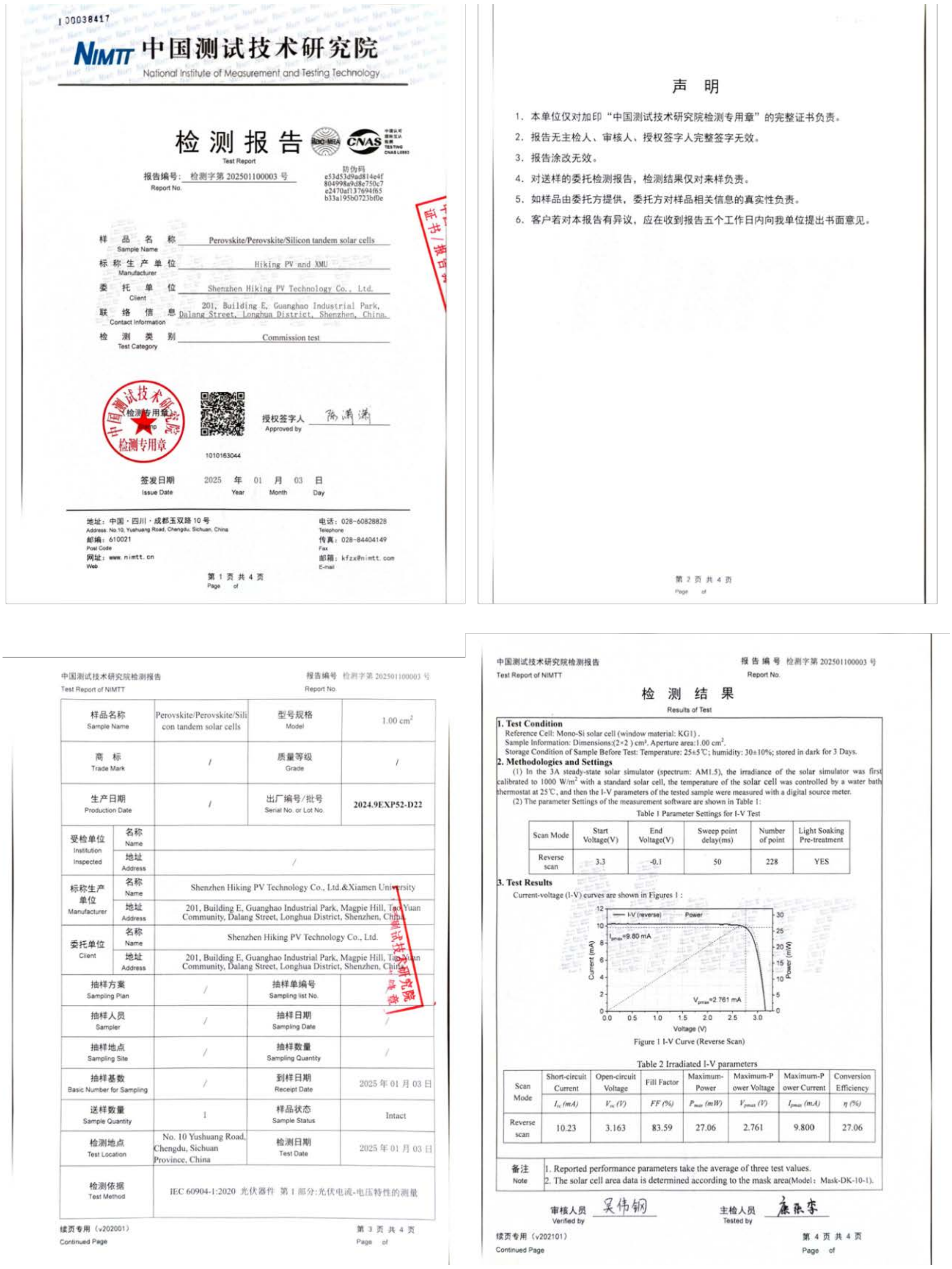

I 00038417

NIMTT 中国测试技术研究院

National Institute of Measurement and Testing Technology

检 测 报 告

Test Report

报告编号：检测字第 202501100003 号

Report No.

防伪码
e53d53d9ad814e4f
804998a9d8e750c7
e2470af137694f65
b33a195b0723bf0e

样品名称 Sample Name: Perovskite/Perovskite/Silicon tandem solar cells

标称生产单位 Manufacturer: Hiking PV and XMU

委托单位 Client: Shenzhen Hiking PV Technology Co., Ltd.

联络信息 Contact Information: 201, Building E, Guanghao Industrial Park, Dalang Street, Longhua District, Shenzhen, China.

检测类别 Test Category: Commission test

中国测试技术研究院（检测专用章）Stamp 检测专用章

1010163044

授权签字人 Approved by: 陈满满

签发日期 Issue Date: 2025 年 Year 01 月 Month 03 日 Day

地址：中国・四川・成都玉双路 10 号
Address: No.10, Yushuang Road, Chengdu, Sichuan, China
邮编：610021
Post Code
网址：www.nimtt.cn
Web

电话：028-60828828
Telephone
传真：028-84404149
Fax
邮箱：kfzx@nimtt.com
E-mail

第 1 页 共 4 页
Page of

声 明

1. 本单位仅对加印“中国测试技术研究院检测专用章”的完整证书负责。
2. 报告无主检人、审核人、授权签字人完整签字无效。
3. 报告涂改无效。
4. 对送样的委托检测报告，检测结果仅对来样负责。
5. 如样品由委托方提供，委托方对样品相关信息的真实性负责。
6. 客户若对本报告有异议，应在收到报告五个工作日内向我单位提出书面意见。

第 2 页 共 4 页
Page of

中国测试技术研究院检测报告 Test Report of NIMTT　　报告编号 检测字第 202501100003 号 Report No.

样品名称 Sample Name		Perovskite/Perovskite/Silicon tandem solar cells	型号规格 Model	1.00 cm²
商标 Trade Mark		/	质量等级 Grade	/
生产日期 Production Date		/	出厂编号/批号 Serial No. or Lot No.	**2024.9EXP52-D22**
受检单位 Institution Inspected	名称 Name	/		
	地址 Address			
标称生产单位 Manufacturer	名称 Name	Shenzhen Hiking PV Technology Co., Ltd.&Xiamen University		
	地址 Address	201, Building E, Guanghao Industrial Park, Magpie Hill, Tao Yuan Community, Dalang Street, Longhua District, Shenzhen, China		
委托单位 Client	名称 Name	Shenzhen Hiking PV Technology Co., Ltd.		
	地址 Address	201, Building E, Guanghao Industrial Park, Magpie Hill, Tao Yuan Community, Dalang Street, Longhua District, Shenzhen, China		
抽样方案 Sampling Plan		/	抽样单编号 Sampling list No.	
抽样人员 Sampler		/	抽样日期 Sampling Date	/
抽样地点 Sampling Site		/	抽样数量 Sampling Quantity	/
抽样基数 Basic Number for Sampling		/	到样日期 Receipt Date	2025 年 01 月 03 日
送样数量 Sample Quantity		1	样品状态 Sample Status	Intact
检测地点 Test Location		No. 10 Yushuang Road, Chengdu, Sichuan Province, China	检测日期 Test Date	2025 年 01 月 03 日
检测依据 Test Method		IEC 60904-1:2020 光伏器件 第 1 部分:光伏电流-电压特性的测量		

续页专用（v202001）Continued Page　　第 3 页 共 4 页 Page of

中国测试技术研究院检测报告 Test Report of NIMTT　　报告编号 检测字第 202501100003 号 Report No.

检 测 结 果

Results of Test

1. Test Condition

Reference Cell: Mono-Si solar cell (window material: KG1).
Sample Information: Dimensions:(2×2) cm². Aperture area:1.00 cm².
Storage Condition of Sample Before Test: Temperature: 25±5℃; humidity: 30±10%; stored in dark for 3 Days.

2. Methodologies and Settings

(1) In the 3A steady-state solar simulator (spectrum: AM1.5), the irradiance of the solar simulator was first calibrated to 1000 W/m² with a standard solar cell, the temperature of the solar cell was controlled by a water bath thermostat at 25℃, and then the I-V parameters of the tested sample were measured with a digital source meter.

(2) The parameter Settings of the measurement software are shown in Table 1:

Table 1 Parameter Settings for I-V Test

Scan Mode	Start Voltage(V)	End Voltage(V)	Sweep point delay(ms)	Number of point	Light Soaking Pre-treatment
Reverse scan	3.3	-0.1	50	228	YES

3. Test Results

Current-voltage (I-V) curves are shown in Figures 1:

Figure 1 I-V Curve (Reverse Scan)

Table 2 Irradiated I-V parameters

Scan Mode	Short-circuit Current	Open-circuit Voltage	Fill Factor	Maximum-Power	Maximum-Power Voltage	Maximum-Power Current	Conversion Efficiency
	I_{sc} (mA)	V_{oc} (V)	FF (%)	P_{max} (mW)	V_{pmax} (V)	I_{pmax} (mA)	η (%)
Reverse scan	10.23	3.163	83.59	27.06	2.761	9.800	27.06

备注 Note: 1. Reported performance parameters take the average of three test values. 2. The solar cell area data is determined according to the mask area(Model：Mask-DK-10-1).

审核人员 Verified by: 吴伟钢　　主检人员 Tested by: 康兆李

续页专用（v202101）Continued Page　　第 4 页 共 4 页 Page of

Supplementary figure 24 Third party verified results of 1.0 cm^2 perovskite-perovskite-silicon triple junction tandem solar cell measured by National Institute of Measurement and Testing Technology (NIMTT), China. The solar cell is measured by the *I*-*V* scan method.

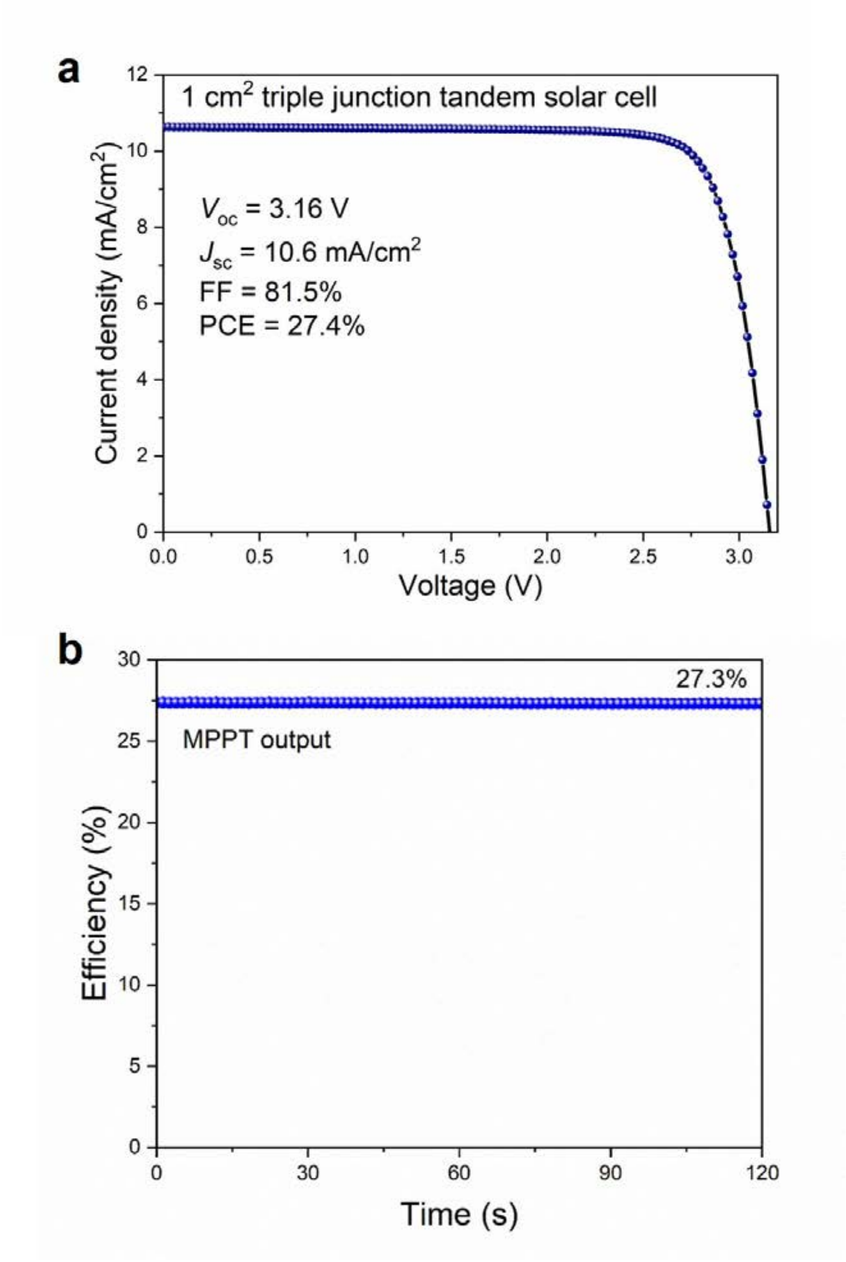

Supplementary figure 25 In-house measured (a) J-V curve and (b) steady state PCE of the 1 cm^2 triple junction champion device.

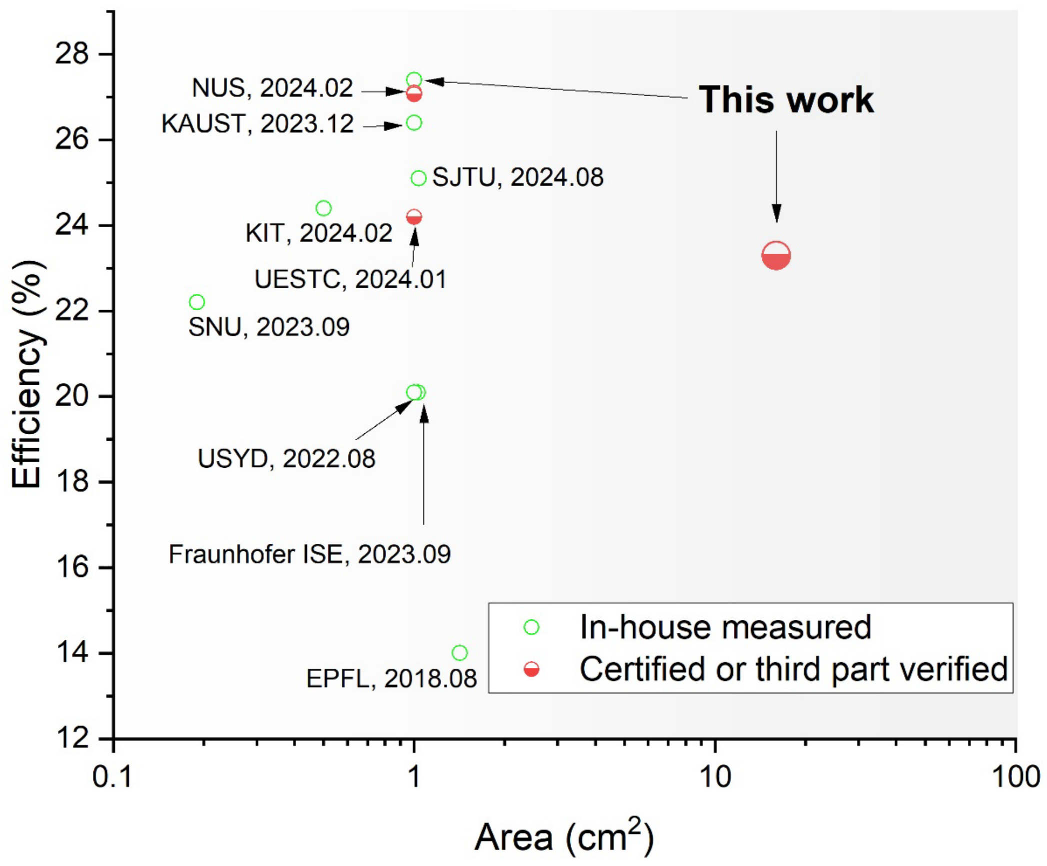

Supplementary figure 26 PCE and device area comparisons of reported perovskite-perovskite-silicon tandem solar cells.

中国认可
国际互认
检测
TESTING
CNAS L8490

230017163974

Test and Calibration Center of New Energy Device and Module,
Shanghai Institute of Microsystem and Information Technology,
Chinese Academy of Sciences (SIMIT)

Measurement Report

Report No. 24TR083001R1

**Client Name**	Shenzhen Hiking PV Technology Co., Ltd. & School of Electronic Science and Engineering, Xiamen University
**Client Address**	502, Building B, Dajiahao Plaza, No.362 Yu'an 2nd Rd, Xin'an Subdistrict, Bao'an District, Shenzhen & 4221, Xiang'an South Road, Xiang'an District, Xiamen, Fujian
**Sample**	Perovskite/perovskite/silicon tandem solar cell
**Manufacturer**	Shenzhen Hiking PV Technology Co., Ltd. & School of Electronic Science and Engineering, Xiamen University
**Measurement Date**	30th August, 2024

**Performed by:**	Qiang Shi	**Date:** 30/08/2024
**Reviewed by:**	Wenjie Zhao	**Date:** 30/08/2024
**Approved by:**	Yucheng Liu	**Date:** 30/08/2024

Address: No.235 Chengbei Road, Jiading, Shanghai **Post Code:**201800

E-mail: solarcell@mail.sim.ac.cn **Tel:** +86-021-69976905

The measurement report without signature and seal are not valid.
This report shall not be reproduced, except in full, without the approval of SIMIT.

Sample Information

Sample Type	Perovskite/perovskite/silicon tandem solar cell
Serial No.	R5
Lab Internal No.	24083001-1#
Measurement Item	I-V characteristic
Measurement Environment	24.9±2.0°C, 51.1±5.0%R.H

Measurement of I-V characteristic

**Reference cell**	PVM1121
**Reference cell Type**	mono-Si, WPVS, calibrated by NREL (Certificate No. ISO 2098)
**Calibration Value/Date of Calibration for Reference cell**	143.95mA/ Feb. 2024
**Measurement Conditions**	Standard Test Condition (STC):<br>Spectral Distribution: AM1.5,<br>Irradiance: 1000±50W/m^2, Temperature: 25±2°C
**Measurement Equipment/ Date of Calibration**	AAA Steady State Solar Simulator (YSS-T155-2M) / Sep.2023<br>IV test system (ADCMT 6246) / June. 2024<br>Measuring Microscope (MF-B2017C) / July.2024<br>Spectral Response Measurement System (CEP-25ML-CAS) / May.2024
**Measurement Method**	I-V Measurement:<br>Logarithmic sweep in both directions (Voc to Isc and Isc to Voc) during one flash based on IEC 60904-1:2020;<br>Spectral Mismatch factor was calculated and I-V correction was performed according to IEC 60891.<br>MPP-Tracking:<br>Tracking for 300 seconds by P&O method, the reported Pmax represents the average value of the total data.
**Measurement Uncertainty**	Area: 1.0%(k=2); Isc: 2.4%(k=2); Voc: 1.0%(k=2);<br>Pmax: 2.9%(k=2); Eff: 3.0%(k=2)

====Measurement Results ====

	Forward Scan (Isc to Voc)		Reverse Scan (Voc to Isc)		MPP-Tracking	
Area			16.0007 cm^2			
Isc	157.310	mA	157.374	mA	/	
Voc	3.050	V	3.063	V	/	
Pmax	368.163	mW	378.492	mW	372.876	mW
Ipm	141.870	mA	143.189	mA	144.142	mA
Vpm	2.595	V	2.643	V	2.587	V
FF	76.73	%	78.51	%	/	
Eff	23.01	%	23.65	%	23.30	%

- Spectral Mismatch Factor SMM_{top}=1.0044, SMM_{mid}=1.0060, SMM_{bot}=1.0056.
- Designated illumination area defined by a thin mask was measured by measuring microscope.
- Test results listed in this measurement report refer exclusively to the mentioned test sample.
- The results apply only at the time of the test, and do not imply future performance.

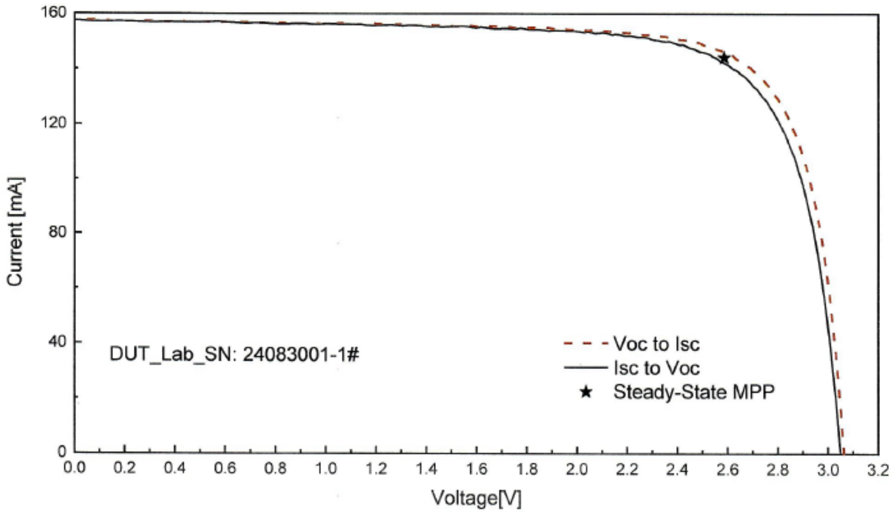

Fig.1 I-V curves of the measured sample

Report No. 24TR083001R1

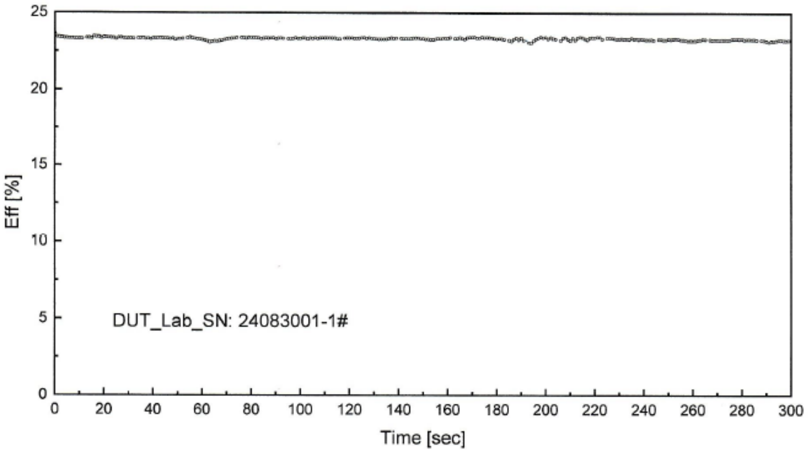

Fig.2 Steady-state maximum power output of the measured sample

-------End of Report-------

4 / 4

Supplementary figure 27 Certified results of 16.0 cm^2 perovskite-perovskite-silicon triple junction tandem solar cell measured by Shanghai Institute of Microsystem and Information Technology, Chinese Academy of Sciences (SIMIT). The solar cell is measured by the *I*-*V* scan method.

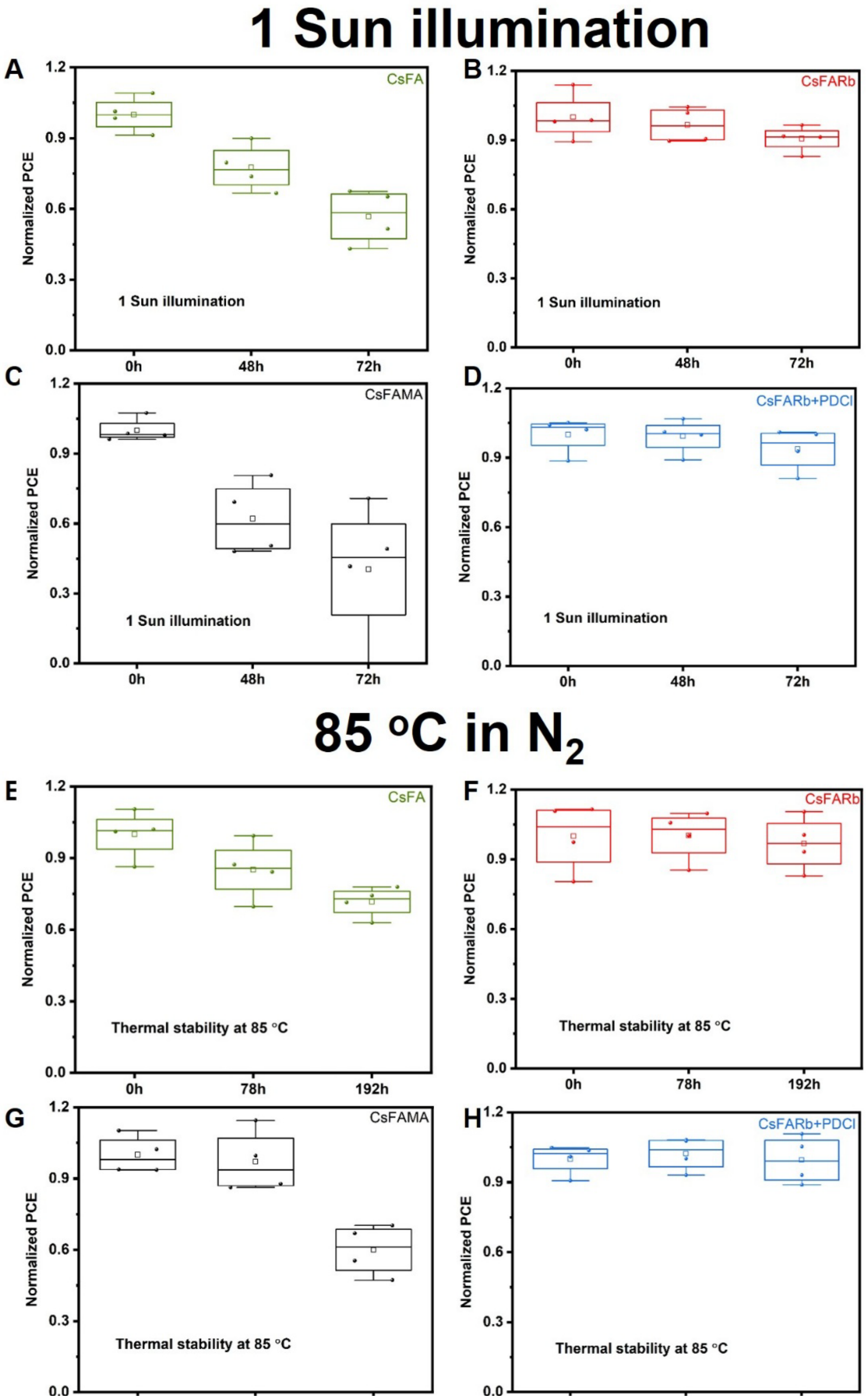

Supplementary figure 28 Stability of encapsulated high bandgap cells with different perovskite compositions under (a-d) continuous 1 sun (Xe lamp) illumination and (e-h) 85°C in N_2 (measured ex-situ). CsFA refers to MA-free and Rb-free $Cs_{0.2}FA_{0.8}Pb(I_{0.45}Br_{0.55})_3$ perovskite. CsFARb refers to MA-free Rb containing $Cs_{0.16}Rb_{0.04}FA_{0.8}Pb(I_{0.45}Br_{0.55})_3$ perovskite, CsFAMA refers to MA-containing Rb-free $Cs_{0.16}MA_{0.04}FA_{0.8}Pb(I_{0.45}Br_{0.55})_3$ perovskite and CsFARb+PDCl refers to

$Cs_{0.16}Rb_{0.04}FA_{0.8}Pb(I_{0.45}Br_{0.55})_3$ perovskite with PDCl treatment (6 devices in each type. Top value: maximum; top bar: 75th percentile; middle bar: median; open squares: mean; bottom bar: 25th percentile; lowest value: minimum. Solid dots: data points).

Supplementary table 1 Summaries of monolithic triple-junction perovskite-perovskite-Si tandems

Pero-Si Interface	Mid bandgap	Pero-pero Interface	High bandgap	V_{OC} (V)	J_{SC} (mA/cm^2)	FF (%)	Eff. (%)	Area (cm^2)	Stability	Ref.
nc-Si:H(p+)/ nc-Si:H(n+)	Spiro-TTB/1.53 eV $Cs_xFA_{1-x}Pb(I,Br)_3$ /LiF/C_{60}/ALD-SnO_2	IZO (150 nm)	NiO_X/1.8 eV $Cs_xFA_{1-x}Pb(I,Br)_3$ /LiF/C_{60}/ALD-SnO_2/IZO	2.69	7.7	68	14.0	1.4	Unencapsulated – 118% of initial PCE after 1250s of MPPT	1
				2.41	9.6	56	13.0	1.4		
ITO	MeO-2PACz/1.55 eV $Cs_{0.1}FA_{0.9}PbI_3$/LiF/C_{60}/ALD-SnO_2	Au (1 nm)	NiO_X/MeO-2PACz/1.90 eV $Cs_{0.2}FA_{0.8}Pb(I_{0.45}Br_{0.55})I_3$/LiF/$C_{60}$/ALD-$SnO_2$/ITO	2.74	8.54	86	20.1	1.0	Unencapsulated – 100.4% of initial PCE after 44s of fixed voltage	2
ITO	PTAA/1.56eV $Cs_{0.1}FA_{0.9}PbI_3$/C_{60}/PEIE	ITO (20 nm)	Me-4PACz/1.96eV $MAPb(I_{0.5}Br_{0.35}Cl_{0.15})_3$/$C_{60}$/PEIE/ITO	2.78	10.2	79	22.2	0.2	Unencapsulated – 99% of initial PCE after 600s of fixed voltage	3
ITO	PTAA/1.56eV $Cs_{0.05}(FA_{0.9}MA_{0.1})_{0.95}Pb(I_{0.95}Br_{0.05})_3$/$C_{60}$/$SnO_X$	ITO (15 nm)	2PACz/1.83eV $Cs_{0.05}(FA_{0.55}MA_{0.45})_{0.95}Pb(I_{0.55}Br_{0.45})_3$/$C_{60}$/$SnO_X$/ITO	2.87	8.9	78	20.1	1.0	Not reported	4
IZO	MeO-2PACz/1.52eV $Rb_{0.05}Cs_{0.1}FA_{0.85}PbI_3$/C60/$SnO_2$	IZO (35 nm)	NiOx/2PACz/2.0eV $Cs_{0.1}FA_{0.9}PbBr_{2.1}I_{0.9}$/C60/$SnO_2$/IZO/$MgF_2$	3.04	11.9	73	26.4	1.0	Unencapsulated – 88.5% of initial PCE after 1000h on 85_C hotplate in an N_2-filled glovebox.	5
ITO	NiO_X/2PACz/1.52eV $FAPbI_3$/C_{60}/SnO_X	ITO (15 nm)	NiO_X/1.84eV 2PACz/$Cs_{0.2}FA_{0.8}Pb(I_{0.5}Br_{0.5})$/$C_{60}$/$SnO_X$/IZO	2.84	11.6	74	24.4	0.5	Unencapsulated - 96% of initial PCE after 10h of MPPT<br>Unencapsulated - 97% of initial PCE after 1081h of	6

									storage in the dark at 85°C	
IZO	NiO_X/2PACz/(1.55 eV $Cs_{0.05}(FA_{0.98}MA_{0.02})_{0.95}Pb(I_{0.98}Br_{0.02})_3$/ LiF/$C_{60}$/ALD SnO_X	IZO (60 nm)	NiO_X/2PACz/1.98 eV $Rb_{0.05}Cs_{0.12}FA_{0.83}PbI_{0.95}Cl_{0.05}Br_2$/LiF/$C_{60}$/ALD SnO_X/X	2.97	11.8	69	24.2*	1.0	Unencapsulated - 87% of initial PCE after 100h of MPPT.	7
ITO	Me-4PACz/1.55eV $FA_{0.9}Cs_{0.1}PbI_3/C_{60}/SnO_2$	ITO (8 nm)	NiO_X/Me-4PACz/1.93eV $FA_{0.6}MA_{0.15}Cs_{0.25}Pb(I_{0.45}Br_{0.5}OCN_{0.05})/C_{60}/SnO_2$/ITO/LiF	3.15	11.0	78	27.1*+	1.0	Encapsulated -80% of initial PCE after 300h of MPPT	8
ITO	NiOx/Me-4PACz/1.55eV $Cs_{0.05}(FA_{0.98}MA_{0.02})Pb(I_{0.98}Br_{0.02})_3/C_{60}/SnO_2$	IZO (60 nm)	NiOx/Me-4PACz/1.95eV $Cs_{0.05}FA0_{.85}MA_{0.15})_{0.95}Pb(I_{0.45}Br_{0.55})_3$/MSE/ C_{60}/SnO2/ZIO/MgFx	3.07	11.4	72	25.1	1.0	Unencapsulated - 93% of initial PCE after 280 min of MPPT tracking.	9
**ITO**	**MeO-2PACz/1.55 eV $Cs_{0.08}Rb_{0.02}FA_{0.9}PbI_3/C_{60}$/ALD-$SnO_2$**	**Au nanoparticles (~3.2nm ∅)**	**NiO_X/MeO-2PACz/1.91 eV $Cs_{0.16}Rb_{0.04}FA_{0.8}Pb(I_{0.45}Br_{0.55})I_3$/PDCl/$C_{60}$/ ALD-$SnO_2$/ITO/$MgF_2$**	**3.16**	**10.6**	**82**	**27.4**	**1.0**	**Encapsulated (device area: $1cm^2$) - 95% of initial PCE after 407h of MPPT**	**This work**
				**3.16**	**10.2**	**84**	**27.06***	**1.0**		
				**3.06**	**9.8**	**79**	**23.3*+**	**16.0**	**Encapsulated (device area: $1cm^2$) - 97% of initial PCE after 200 thermal cycles (40°C ↔85°C)**	

* Certified or third-party verified.

+ Steady-state (5min) power conversion efficiency

Supplementary table 2 Photovoltaic parameters of champion Control and PDCl based single junction high bandgap (1.91 eV) perovskite cells under reverse scan

Device		V_{OC} (mV)	J_{SC} (mA/cm^2)	FF (%)	PCE (%)
**Control**	Average	1280±54	13.9±0.8	78.4±2.1	13.7±1.0
	Champion	1286	14.7	80.0	15.1
**PDCl**	Average	1322±32	13.9±0.8	80.9±2.2	15.1±1.3
	Champion	1324	14.7	84.5	16.4

Supplementary table 3 Bi-exponential fitting results of PL decay traces for 1.91 eV high bandgap perovskite without and with PDCl treatment

Sample	τ1 (ns)	Standard deviation (τ1) (ns)	τ2 (ns)	Standard deviation (τ2) (ns)
**Control**	0.9	0.02	4.43	0.09
**PDCl**	1.46	0.02	7.49	0.06

Supplementary table 4 Photovoltaic parameters of rudimentary perovskite-perovskite semitransparent tandem test cells without Rb incorporation or PDCl treatment with varying Au deposition times

Nominal reading of Au "thickness" during deposition(nm)	J_{SC} (mA/cm^2)	V_{OC} (mV)	FF (%)	PCE (%)
**0**	10.3	2280	60.9	15.9
**0.2**	10.1	2125	77.5	16.7
**0.4**	9.9	2162	79.8	17.1
**0.8**	9.3	2159	78.8	15.8
**1.0**	8.9	2127	81.1	15.4

Supplementary table 5 Au nano-particle coverage on SnO_2 layer determined from TEM images. The nominated thicknesses were values read out from the thermal evaporator thickness monitor as deposition time increased.

Nominated thickness (nm)	Average size (nm)	Coverage (%)	Average spacing (nm)
**"0.0"**	Not applicable		
**"0.2"**	Could not be measured under TEM		
**"0.4"**	3.2	32	3.3
**"0.6"**	3.5	41	2.9
**"0.8"**	4.1	48	2.8
**"1.0"**	4.2	56	2.8
**"1.4"**	5.4	58	2.6
**"1.8"**	Clusters of various sizes instead of particles	62	Varied
**"2.2"**		67	
**"2.6"**		72	
**"3.0"**	Semi-continuous film	70	Semi-continuous film

Reference

(1) Werner, J.; Sahli, F.; Fu, F.; Diaz Leon, J. J.; Walter, A.; Kamino, B. A.; Niesen, B.; Nicolay, S.; Jeangros, Q.; Ballif, C. Perovskite/Perovskite/Silicon Monolithic Triple-junction Solar Cells with a Fully Textured Design. *ACS Energy Letters* **2018**, *3* (9), 2052–2058.

(2) Zheng, J.; Wang, G.; Duan, W.; Mahmud, M. A.; Yi, H.; Xu, C.; Lambertz, A.; Bremner, S.; Ding, K.; Huang, S.; Ho-Baillie, A. W. Y. Monolithic Perovskite–Perovskite–Silicon Triple-Junction Tandem Solar Cell with an Efficiency of over 20%. *ACS Energy Letters* **2022**, 3003-3005.

(3) Choi, Y. J.; Lim, S. Y.; Park, J. H.; Ji, S. G.; Kim, J. Y. Atomic Layer Deposition-Free Monolithic Perovskite/Perovskite/Silicon Triple-Junction Solar Cells. *ACS Energy Letters* **2023**, *8* (7), 3141-3146.

(4) Heydarian, M.; Heydarian, M.; Bett, A. J.; Bivour, M.; Schindler, F.; Hermle, M.; Schubert, M. C.; Schulze, P. S. C.; Borchert, J.; Glunz, S. W. Monolithic Two-Terminal Perovskite/Perovskite/Silicon Triple-Junction Solar Cells with Open Circuit Voltage >2.8 V. *ACS Energy Lett* **2023**, *8* (10), 4186-4192.

(5) Xu, F.; Aydin, E.; Liu, J.; Ugur, E.; Harrison, G. T.; Xu, L.; Vishal, B.; Yildirim, B. K.; Wang, M.; Ali, R.; et al. Monolithic perovskite/perovskite/silicon triple-junction solar cells with cation double displacement enabled 2.0 eV perovskites. *Joule* **2024**, *8* (1), 224-240.

(6) Hu, H.; An, S. X.; Li, Y.; Orooji, S.; Singh, R.; Schackmar, F.; Laufer, F.; Jin, Q.; Feeney, T.; Diercks, A.; et al. Triple-junction perovskite-perovskite-silicon solar cells with power conversion efficiency of 24.4. *Energy Environ Sci* **2024**, *17* (8), 2800-2814.

(7) Li, F.; Wu, D.; Shang, L.; Xia, R.; Zhang, H.; Huang, Z.; Gong, J.; Mao, L.; Zhang, H.; Sun, Y.; et al. Highly Efficient Monolithic Perovskite/Perovskite/Silicon Triple-Junction Solar Cells. *Advanced materials* **2024**, e2311595.

(8) Liu, S.; Lu, Y.; Yu, C.; Li, J.; Luo, R.; Guo, R.; Liang, H.; Jia, X.; Guo, X.; Wang, Y.-D.; et al. Triple-junction solar cells with cyanate in ultrawide-bandgap perovskites. *Nature* **2024**, *628* (8007), 306-312.

(9) Ye, T.; Qiao, L.; Wang, T.; Wang, P.; Zhang, L.; Sun, R.; Kong, W.; Xu, M.; Yan, X.; Yang, J.; et al. Molecular Synergistic Effect for High Efficiency Monolithic Perovskite/Perovskite/Silicon Triple - Junction Tandem Solar Cells. *Advanced Energy Materials* **2024**, 2402491.
