# Supplementary material for: Tailoring nanoscale interfaces for perovskite–perovskite–silicon triple-junction solar cells
Source: Nat Nanotechnol. 2025 Oct 7;20(11):1648–55. doi: 10.1038/s41565-025-02015-x (PMC12623231; doi:10.1038/s41565-025-02015-x)
Supplement: Supplementary file 2 — Reporting Summary [file 41565_2025_2015_MOESM2_ESM.pdf]

nature research

Corresponding author(s): Anita Ho-Baillie, Zhong Chen, Leiping Duan, Jianghui Zheng

Solar Cells Reporting Summary

Nature Research wishes to improve the reproducibility of the work that we publish. This form is intended for publication with all accepted papers reporting the characterization of photovoltaic devices and provides structure for consistency and transparency in reporting. Some list items might not apply to an individual manuscript, but all fields must be completed for clarity.

For further information on Nature Research policies, including our data availability policy, see Authors & Referees.

Experimental design

Please check: are the following details reported in the manuscript?

1. Dimensions

Item	Yes/No	Details
Area of the tested solar cells	☒ Yes ☐ No	0.0706 cm2 aperture for single junction perovskite devices when tested in house<br>0.09 cm2 aperture for semitransparent double junction perovskite devices when tested in house<br>1.0 cm2 and 16.0cm2 apertures for perovskite-perovskite-Si triple junction devices when tested in house and by third party.
Method used to determine the device area	☒ Yes ☐ No	Aperture

2. Current-voltage characterization

Item	Yes/No	Details
Current density-voltage (J-V) plots in both forward and backward direction	☐ Yes ☒ No	Reverse scan only (from near VOC to JSC) when done in house and at NIMTT<br>Both directions when done SiMIT
Voltage scan conditions<br>*For instance: scan direction, speed, dwell times*	☒ Yes ☐ No	Single and double junction devices were measured with a scan rate of 100 mV/s in reverse scan at room temperature in ambient air when done in house.<br>Triple junction devices were measured with a scan rate of 100 mV/s in reverse scan at room temperature in ambient air when done in house.<br>1cm2 triple junction devices were measured with a sweep point delay of 50s for 228 data points in reverse scan at controlled 25 degrees Celsius in ambient air when done in NIMTT.<br>16cm2 triple junction devices were measured at controlled 25 degrees Celsius at 51% relative humidity when done in SiMIT.
Test environment<br>*For instance: characterization temperature, in air or in glove box*	☒ Yes ☐ No	See above
Protocol for preconditioning of the device before its characterization	☒ Yes ☐ No	No precondition before testing when done in-house<br>Light soaking pretreatment can be supplied by NIMTT when requested
Stability of the J-V characteristic<br>*Verified with time evolution of the maximum power point or with the photocurrent at maximum power point; see ref. 7 for details.*	☒ Yes ☐ No	In-house steady state output measurement of the 1cm2 triple junction is in Figure S25b (120 seconds).<br>SiMIT measured steady state output for the 16cm2 triple junction is in Figure S27 (300 seconds).

3. Hysteresis or any other unusual behaviour

Item	Yes/No	Details
Description of the unusual behaviour observed during the characterization	☐ Yes ☒ No	We did not observe unusual behaviour during measurement
Related experimental data	☒ Yes ☐ No	For 16cm2 measured results, please see Figure S27

4. Efficiency

Item	Yes/No	Details
External quantum efficiency (EQE) or incident photons to current efficiency (IPCE)	☒ Yes ☐ No	Figure S5 for 1.91eV single junction perovskite cell<br>Figure 4c for triple junction tandem solar cell
A comparison between the integrated response under the standard reference spectrum and the response measure under the simulator	☒ Yes ☐ No	Good agreement between integrated Jsc from EQE Jsc from J-V measurement.

For tandem solar cells, the bias illumination and bias voltage used for each subcell	☒ Yes ☐ No	Bias illumination was used for EQE measurements of triple junctions and details can be found in the Characterization section in the Supporting Information (SI)

5. Calibration

Light source and reference cell or sensor used for the characterization	☒ Yes ☐ No	Detail information can be found in the Characterization section in the SI for in-house measurements.<br>Detail information on this from NIMTT and SiMIT can be supplied when requested.
Confirmation that the reference cell was calibrated and certified	☒ Yes ☐ No	Detail information can be found in the Characterization section in the SI for in-house measurements.<br>Detail information on this from NIMTT and SiMIT can be supplied when requested.
Calculation of spectral mismatch between the reference cell and the devices under test	☐ Yes ☒ No	No spectral mismatch calculation was performed.

6. Mask/aperture

Size of the mask/aperture used during testing	☒ Yes ☐ No	See answers to Q1 above
Variation of the measured short-circuit current density with the mask/aperture area	☐ Yes ☒ No	Aperture always used

7. Performance certification

Identity of the independent certification laboratory that confirmed the photovoltaic performance	☒ Yes ☐ No	Independent certification/ verifications conducted at NIMTT and SIMIT for 1 cm² and 16 cm2, triple junctions respectively.
A copy of any certificate(s)<br>*Provide in Supplementary Information*	☒ Yes ☐ No	Figure S24 for 1.0 cm2 triple junction solar cell<br>Figure S27 for 16.0 cm2 triple junction solar cell

8. Statistics

Number of solar cells tested	☒ Yes ☐ No	10 devices in Figure 1<br>10 devices in Figure S6<br>10 devices in Figure S9<br>6 devices in Figure S23
Statistical analysis of the device performance	☒ Yes ☐ No	Figures 1b-e; Figures S6b-e; Figures S9a-b; Figure S23a-d

9. Long-term stability analysis

Type of analysis, bias conditions and environmental conditions<br>*For instance: illumination type, temperature, atmosphere humidity, encapsulation method, preconditioning temperature*	☒ Yes ☐ No	International Electrotechnical Commission (IEC) 61215 Thermal Cycling test of encapsulated 1cm2 triple junction device for 200 cycles<br>Maximum power point tracking for 1cm2 triple junction device for 407 house in Figure 4g.<br>1 Sun illumination of encapsulated 1.91eV single junction devices for 72 hours in Figures S28a-d<br>85 degrees Celsius thermal stability of 1.91eV single junction devices for 192 hours in N2 in Figures S28e-h
